# Inhibition of insulin/IGF-1 receptor signaling protects from mitochondria-mediated kidney failure

**DOI:** 10.15252/emmm.201404916

**Published:** 2015-02-02

**Authors:** Christina Ising, Sybille Koehler, Sebastian Brähler, Carsten Merkwirth, Martin Höhne, Olivier R Baris, Henning Hagmann, Martin Kann, Francesca Fabretti, Claudia Dafinger, Wilhelm Bloch, Bernhard Schermer, Andreas Linkermann, Jens C Brüning, Christine E Kurschat, Roman-Ulrich Müller, Rudolf J Wiesner, Thomas Langer, Thomas Benzing, Paul Thomas Brinkkoetter

**Affiliations:** 1 Department II of Internal Medicine and Center for Molecular Medicine Cologne (CMMC), University of CologneCologne, Germany; 2 Institute for Genetics, University of CologneCologne, Germany; 3 Howard Hughes Medical Institute, University of California BerkeleyBerkeley, CA, USA; 4 Cologne Cluster of Excellence on Cellular Stress Responses in Ageing-Associated Diseases (CECAD) and Systems Biology of Ageing Cologne (Sybacol), University of CologneCologne, Germany; 5 Center for Physiology and Pathophysiology, Institute for Vegetative Physiology, University of CologneCologne, Germany; 6 Department of Molecular and Cellular Sport Medicine, Institute of Cardiovascular Research and Sport Medicine, German Sport University CologneCologne, Germany; 7 Division of Nephrology and Hypertension, Christian-Albrechts-UniversityKiel, Germany; 8 Max Planck Institute for Metabolism ResearchCologne, Germany; 9 Center for Endocrinology, Diabetes and Preventive Medicine (CEDP), University Hospital CologneCologne, Germany

**Keywords:** insulin, mitochondria, mTOR, podocyte

## Abstract

Mitochondrial dysfunction and alterations in energy metabolism have been implicated in a variety of human diseases. Mitochondrial fusion is essential for maintenance of mitochondrial function and requires the prohibitin ring complex subunit prohibitin-2 (PHB2) at the mitochondrial inner membrane. Here, we provide a link between PHB2 deficiency and hyperactive insulin/IGF-1 signaling. Deletion of PHB2 in podocytes of mice, terminally differentiated cells at the kidney filtration barrier, caused progressive proteinuria, kidney failure, and death of the animals and resulted in hyperphosphorylation of S6 ribosomal protein (S6RP), a known mediator of the mTOR signaling pathway. Inhibition of the insulin/IGF-1 signaling system through genetic deletion of the insulin receptor alone or in combination with the IGF-1 receptor or treatment with rapamycin prevented hyperphosphorylation of S6RP without affecting the mitochondrial structural defect, alleviated renal disease, and delayed the onset of kidney failure in PHB2-deficient animals. Evidently, perturbation of insulin/IGF-1 receptor signaling contributes to tissue damage in mitochondrial disease, which may allow therapeutic intervention against a wide spectrum of diseases.

## Introduction

The mitochondrial prohibitin (PHB) protein complex comprises two subunits, PHB1 and PHB2, that assemble into a high molecular weight ring complex in the mitochondrial inner membrane (Back *et al*, 2002; Tatsuta *et al*, 2005; Merkwirth & Langer, 2009). PHB proteins have multiple functions, they modulate mitochondrial m-AAA protease activity (Steglich *et al*, 1999) and control lipid distribution in the mitochondrial inner membrane (Osman *et al*, 2009b), and they serve as membrane-bound chaperones for the assembly of mitochondrial-encoded proteins (Nijtmans *et al*, 2000) and recruit membrane proteins to a specific lipid environment (Osman *et al*, 2009a). Acting as a membrane scaffold, the PHB complex is involved in maintaining mitochondrial integrity, indispensable for cristae morphogenesis, and fusion of the organelles (Kasashima *et al*, 2008; Merkwirth *et al*, 2008). Cellular depletion of PHB2 in mouse embryonic fibroblasts (MEFs) led to disorganized and swollen mitochondrial cristae structures (Merkwirth *et al*, 2008). The conventional knockout of *Phb2* in a mouse model resulted in embryonic lethality (Park *et al*, 2005; Merkwirth *et al*, 2008) while a conditional, neuron-specific PHB2 deficiency led to neurodegeneration associated with loss of the mitochondrial genome and severe mitochondrial dysfunction (Merkwirth *et al*, 2012).

Deregulation of mitochondrial function, impaired energy homeostasis, and the production of reactive oxygen species have been implicated in aging-associated phenotypes (Nunnari & Suomalainen, 2012; Bratic & Larsson, 2013) and a large variety of human disorders including kidney disease (Imasawa & Rossignol, 2013). The majority of kidney diseases affect the renal filtration unit, the glomerulus, as a consequence of a very limited capacity for regeneration and self-renewal of terminally differentiated glomerular podocytes (Brinkkoetter *et al*, 2013). Podocytes are epithelial cells that have interdigitating foot processes to cover the outer surface of glomerular capillaries at the kidney filtration barrier and form the filtration slits. Podocytes are essential components of the kidney filtration unit and responsible for ultrafiltration of protein-free urine from plasma.

To understand the contribution of mitochondrial dysfunction to kidney disease, we generated podocyte-specific *Phb2* knockout mice. Loss of PHB2 in podocytes inevitably led to progressive proteinuria, glomerulosclerosis, end-stage renal failure, and death of the animals within 4–5 weeks. Surprisingly, either additional knockout of the insulin and IGF-1 receptor or treatment with the mTORC1 inhibitor rapamycin partially rescued the phenotype of *Phb2* knockout mice and prolonged survival.

## Results

### Podocyte-specific *Phb2* knockout mice develop albuminuria and die prematurely

**Figure 1:**
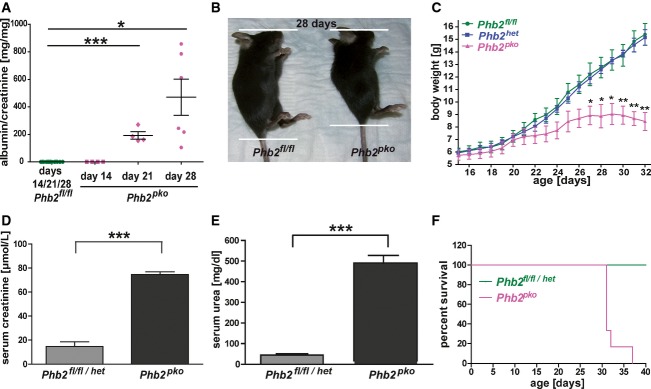
Podocyte-specific *Phb2* knockout mice develop albuminuria and die prematurely
A Measurement of urinary albumin/creatinine by ELISA (albumin-to-creatinine ratio: day 14: *Phb2*^*fl/fl*^ 0.09 ± 0.03 mg/mg, *n *= 4, versus *Phb2*^*pko*^ 0.37 ± 0.21 mg/mg, *n* = 4, *P = *0.2350; day 21: *Phb2*^*fl/fl*^ 0.65 ± 0.16 mg/mg, *n* = 4, versus *Phb2*^*pko*^ 193.10 ± 26.81 mg/mg, *n* = 4, ***P = 0.003; day 28: *Phb2*^*fl/fl*^ 0.57 ± 0.07 mg/mg, *n* = 4, versus *Phb2*^*pko*^ 470.40 ± 131.30 mg/mg, *n* = 4, *P = 0.0117).B Appearance of *Phb2*^*fl/fl*^ and *Phb2*^*pko*^ mice at day 28.C Analysis of body weight from postnatal day 14 until day 32 (*P*-values for *Phb2*^*fl/fl*^ or *Phb2*^*het*^ versus *Phb2*^*pko*^: *P = 0.0372 at day 27, *P = 0.0311 at day 28, *P = 0.0157* *at day 29, **P = 0.008* *at day 30, **P = 0.0013* *at day 31, **P = 0.0035* *at day 32, *n* = 3 for *Phb2*^*fl/fl*^ and *n* = 4 for *Phb2*^*het*^ and *Phb2*^*pko*^).D Measurement of serum creatinine levels of mice in their fifth week of life (*Phb2*^*fl/fl/het*^ 14.74 ± 3.75 μmol/l versus *Phb2*^*pko*^ 74.73 ± 2.20 μmol/l, *n = *3 for both groups; ***P = 0.0002).E Measurement of serum urea levels of mice in their fifth week of life (*Phb2*^*fl/fl/het*^ 44.00 ± 7.02 mg/dl versus *Phb2*^*pko*^ 493.70 ± 45.34 mg/dl, *n *= 3 for both groups; ***P = 0.006).F Kaplan–Meier survival curve (*n* = 5 for *Phb2*^*fl/fl/het*^ and *n* = 6 for *Phb2*^*pko*^).
Data information: Results are presented as means ± SEM. (A, C–E) Student's *t*-test.

To understand the contribution of mitochondrial dysfunction to kidney disease, we deleted the *Phb2* gene specifically in podocytes (*Phb2*^*pko*^) by mating a conditional *Phb2*^*fl/fl*^ mouse line (Merkwirth *et al*, 2008) to podocyte-specific Cre mice (*NPHS2.cre*) (Moeller *et al*, 2003). Mice of all genotypes were born following Mendelian rules (Supplementary Fig S1A). At birth, *Phb2*^*pko*^ mutants appeared as healthy as wild-type or heterozygous controls and did not show signs of glomerular dysfunction. The absence of PHB2 resulted in progressive and marked proteinuria reaching an albumin-to-creatinine ratio of 470 mg/mg at day 28 (Fig1A), growth retardation, and massive loss of body weight in comparison with their wild-type (*Phb2*^*fl/fl*^) and heterozygous littermates (*Phb2*^*het*^) (Fig1B and C). *Phb2*^*pko*^ mutant animals developed renal failure as indicated by a rise in serum creatinine and urea (Fig1D and E). All animals died prematurely within 31–37 days postpartum (Fig1F).

### *Phb2*^*pko*^ mice develop glomerulosclerosis

**Figure 2:**
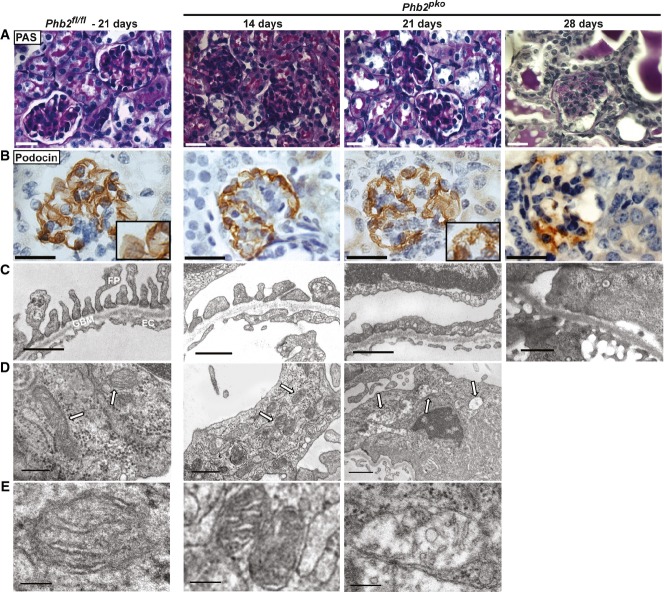
*Phb2*^*pko*^ mice develop glomerulosclerosis
A PAS staining of kidney sections (scale bar: 20 μm).B Immunohistochemistry for podocin on kidney sections (scale bar: 20 μm).C Analysis of podocyte foot processes in electron micrographs (scale bar: 0.7 μm). FP, podocyte foot processes; GBM, glomerular basement membrane; EC, endothelial cells.D Analysis of mitochondrial morphology in electron micrographs (arrows; scale bar: 0.3 μm).E High-power view of mitochondria in electron micrographs (scale bar: 0.1 μm).
Data information: Genotypes and time points as indicated in the figure.

Histological analyses confirmed a normal renal histology at 14 days after birth but revealed progressive glomerulosclerosis at later stages, a phenotype closely resembling human glomerular disease (Fig2A). At 28 days, *Phb2*^*pko*^ mice presented with marked deposition of protein casts in the tubular system, collapsing glomerular capillaries and global glomerulosclerosis.

It is well established that in podocyte disease, the elaborate cytoarchitecture of podocyte foot processes is altered, leading to loss of the typical slit diaphragm structure. Slit diaphragms form a membrane-like cell-to-cell contact that contains a protein complex of podocin and associated proteins (Brinkkoetter *et al*, 2013). Consistent with glomerulosclerosis being the result of podocyte disease, immunohistochemistry stainings for podocin showed alterations at 21 days and a dispersed and reduced staining at 28 days (Fig2B). At 21 days, we observed podocyte effacement, that is, severe changes in foot process organization in all analyzed glomeruli of *Phb2*^*pko*^ mice (Fig2C). Podocyte effacement results in proteinuria, progressive renal damage with glomerular scarring, and eventual loss of renal function, necessitating renal replacement therapy in humans. At 28 days, the podocyte foot process structure was completely lost. As PHB2 is required for maintaining the structure of mitochondrial cristae, we assessed mitochondrial architecture (Fig2D and E). At 14 days, both mitochondrial cristae structures and overall morphology were unaffected. However, 1 week later, lamellar cristae structures were disorganized or completely lost in mitochondria of *Phb2*^*pko*^ podocytes, which is in line with previous reports of mitochondrial changes after deletion of *Phb2* in neurons (Merkwirth *et al*, 2012). At 28 days, we were unable to analyze mitochondrial appearance due to progressive sclerosis and the overall loss of glomerular architecture.

As loss of PHB2 might render podocytes susceptible to apoptosis (Merkwirth *et al*, 2008, 2012; Baris *et al*, 2011; Wu & Wu, 2012), we studied whether the development of albuminuria was due to increased levels of apoptotic cell death resulting in reduced podocyte cell number. At 21 days, staining for cleaved caspase-3 was completely negative (Supplementary Fig S2A). In addition, we quantified podocyte cell number by immunohistochemistry staining for WT-1 in a blinded manner. Again, there were no differences in the overall podocyte cell number between *Phb2*^*fl/fl*^ control and *Phb2*^*pko*^ animals (Supplementary Fig S2B and C). In summary, *Phb2*^*pko*^ mice did not show signs of increased apoptosis or cell loss at this early time point.

### The phenotype of *Phb2*^*pko*^ mice is not developmental

**Figure 3:**
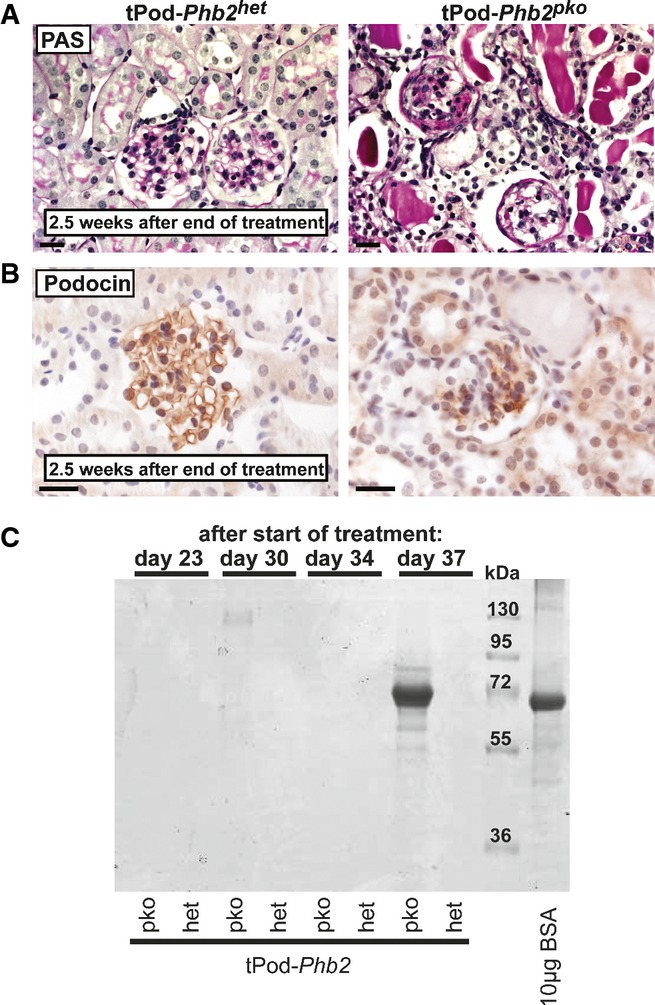
The phenotype of *Phb2*^*pko*^ mice is not developmental
A PAS staining of tPod–*Phb2*^*pko*^ and control mice 2.5 weeks after the end of tamoxifen treatment (scale bar: 20 μm).B Immunohistochemistry for podocin on kidney sections of tPod–*Phb2*^*pko*^ and control mice 2.5 weeks after the end of tamoxifen treatment (scale bar: 20 μm).C Coomassie stain of urinary samples of tPod–*Phb2*^*pko*^ and control mice.

Although all relevant phenotypic changes were observed after day 14, at a time glomerulogenesis is completed, a developmental phenotype resulting from PHB2 loss could still not be excluded. We therefore tested whether the induction of PHB2 loss in adulthood might cause a similar phenotype. *Phb2*^*fl/fl*^ mice were mated to podocin-iCreER(T2) mice that allow expression of Cre recombinase activity upon tamoxifen induction (Wang *et al*, 2010). The resulting podocin-iCreER(T2) *Phb2*^*pko*^ animals (tPod–*Phb2*^*pko*^) received a tamoxifen-enriched diet and developed progressive glomerulosclerosis (Fig3A and B) and massive albuminuria (Fig3C) as observed in non-inducible *Phb2*^*pko*^ mice. Thus, the phenotype of *Phb2*^*pko*^ mice does not appear to be the result of altered developmental programs.

### Podocyte-specific knockout of the insulin receptor and IGF-1 receptor prolongs survival of *Phb2*^*pko*^ mice

**Figure 4:**
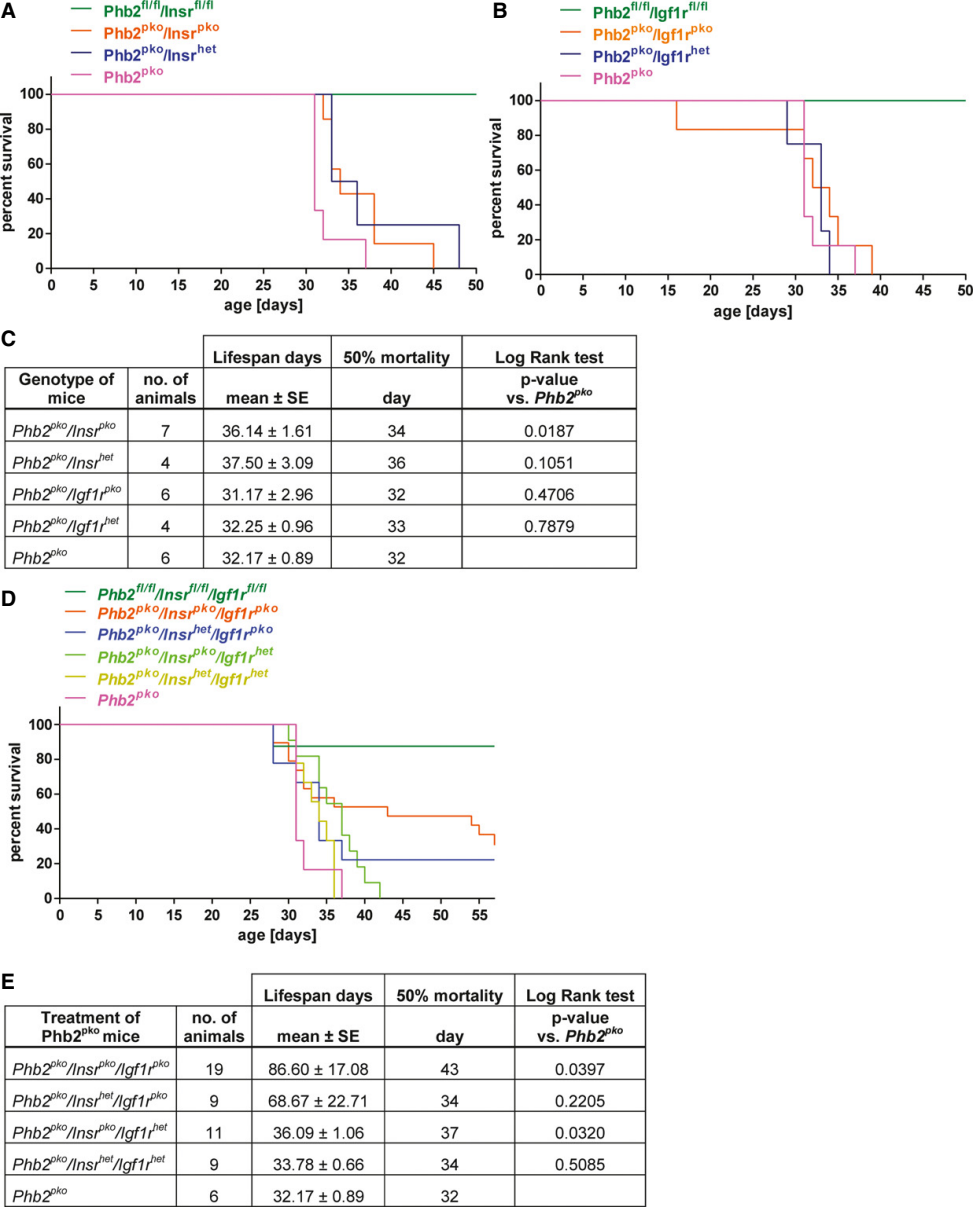
Podocyte-specific knockout of the insulin receptor and IGF-1 receptor prolongs survival of *Phb2*^*pko*^ mice
A Kaplan–Meier survival curve showed that an additional knockout of the insulin receptor (*Insr*) prolonged survival of *Phb2*^*pko*^ mice (*n* = 7 for *Phb2*^*pko*^*/Insr*^*pko*^, *n* = 4 for *Phb2*^*pko*^*/Insr*^*het*^, and *n* = 6 for *Phb2*^*pko*^).B Kaplan–Meier survival curve revealed that the survival time of *Phb2*^*pko*^ mice is not changed by an additional *Igf1r* deficiency (*n* = 6 for *Phb2*^*pko*^*/Igf1r*^*pko*^, *n* = 4 for *Phb2*^*pko*^*/Igf1r*^*het*^, and *n* = 6 for *Phb2*^*pko*^).C Statistical analysis comparing all genotypes with *Phb2*^*pko*^ mice.D Kaplan–Meier survival curve revealed prolonged survival of *Phb2*^*pko*^*/Insr*^*pko*^*/Igf1r*^*pko*^ and *Phb2*^*pko*^*/Insr*^*pko*^*/Igf1r*^*het*^ mice (*n* = 19 for *Phb2*^*pko*^*/Insr*^*pko*^*/Igf1r*^*pko*^, *n* = 9 for *Phb2*^*pko*^*/Insr*^*het*^*/Igf1r*^*pko*^, *n* = 11 for *Phb2*^*pko*^*/Insr*^*pko*^*/Igf1r*^*het*^, *n* = 9 for *Phb2*^*pko*^*/Insr*^*het*^*/Igf1r*^*het*^, and *n* = 6 for *Phb2*^*pko*^).E Statistical analysis comparing all genotypes with *Phb2*^*pko*^ mice.

**Figure 5:**
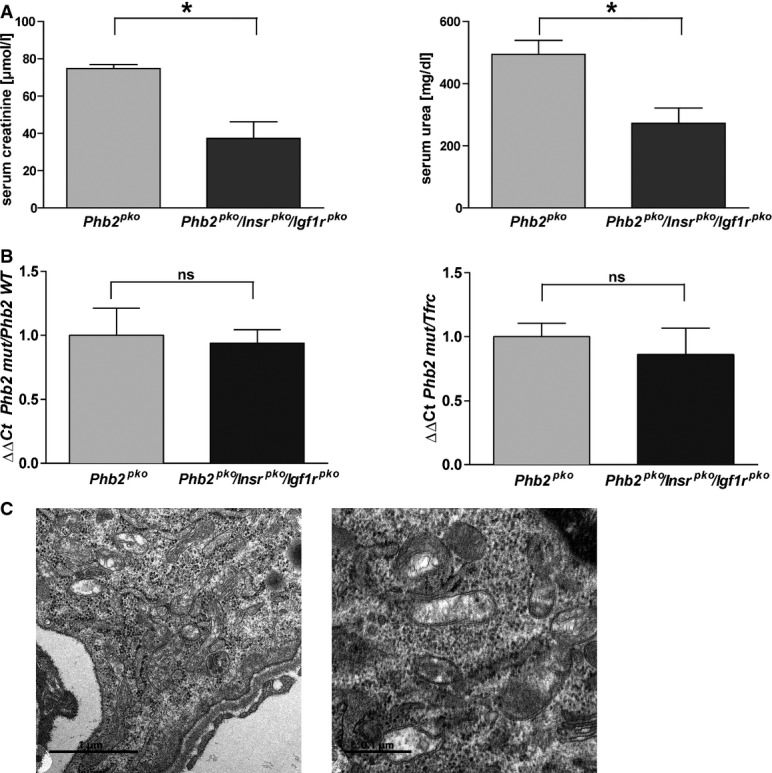
Loss of the insulin and IGF-1 receptor from *Phb2*^*pko*^ mice does not improve mitochondrial ultrastructure
A Serum creatinine and urea levels in the fifth week of life (left graph: *Phb2*^*pko*^ 74.73 ± 2.20 μmol/l versus *Phb2*^*pko*^*/Insr*^*pko*^*/Igf1r*^*pko*^ 37.36 ± 8.83 μmol/l, *n = *3 for *Phb2*^*pko*^ and *n *= 4 for *Phb2*^*pko*^*/Insr*^*pko*^*/Igf1r*^*pko*^, *P = 0.0169; right graph: *Phb2*^*pko*^ 493.70 ± 45.34 mg/dl versus *Phb2*^*pko*^*/Insr*^*pko*^*/Igf1r*^*pko*^ 272.80 ± 49.29 mg/dl, *n *= 3 for *Phb2*^*pko*^ and *n =* 4 for *Phb2*^*pko*^*/Insr*^*pko*^*/Igf1r*^*pko*^, *P = 0.0247).B Abundance of deleted *Phb2* gene in isolated glomeruli of *Phb2*^*fl/fl*^, *Phb2*^*het*^, *Phb2*^*pko*^ and *Phb2*^*pko*^*/Insr*^*pko*^*/Igf1r*^*pko*^ mice compared to the floxed *Phb2* gene (left graph; quantified by qPCR: *n* = 3 for all groups, *P* = 0.83 for *Phb2*^*pko*^*/Insr*^*pko*^*/Igf1r*^*pko*^ versus *Phb2*^*pko*^) and compared to a reference gene (right graph; quantified by qPCR: *n* = 3 for all groups, *P* = 0.58 for *Phb2*^*pko*^*/Insr*^*pko*^*/Igf1r*^*pko*^ versus *Phb2*^*pko*^).C Analysis of mitochondrial morphology in electron micrographs (left image: scale bar, 1 μm; right image: scale bar, 0.1 μm).
Data information: Results are presented as means ± SEM. Student's *t*-test.

Studies in yeast and the nematode *Caenorhabditis elegans* provided an intriguing link of mitochondrial PHB deficiency and altered insulin signaling (Artal-Sanz & Tavernarakis, 2009; Schleit *et al*, 2013). In worms, loss of PHB2 decreased survival but prolonged lifespan of long-lived *daf-2* mutants (Artal-Sanz & Tavernarakis, 2009). In yeast, *Phb2* deficiency activated the mitochondrial unfolded protein response (mtUPR), which was associated with reduced lifespan. This effect could be inhibited by dietary restriction reducing the mtUPR (Schleit *et al*, 2013). Given the complex, yet ill-defined interactions of *Phb2* deficiency and insulin signaling in model organisms, we tested whether inhibition of insulin and/or IGF-1 receptor signaling specifically in podocytes modulated latency or severity of the disease phenotype of *Phb2*^*pko*^ animals. Surprisingly, genetic deletion of the insulin receptor (*Insr*) in podocytes but not of the IGF-1 receptor (*Igf1r*) attenuated renal disease of *Phb2*^*pko*^ mice (Fig4A and B). *Phb2*^*pko*^ mice lacking a functional insulin receptor in podocytes (*Insr*^*pko*^) showed a highly significant survival advantage (Fig4C). Generation of triple-knockout animals with a genetic deletion of both insulin receptor and IGF-1 receptor in addition to PHB2 (*Phb2*^*pko*^*/Insr*^*pko*^*/Igf1r*^*pko*^) significantly alleviated renal disease, enhanced survival (Fig4D and E), improved kidney function, and delayed the onset of kidney failure (Fig5A).

To exclude the possibility that the Cre recombinase insufficiently recombines three genes and thereby alleviates the Phb2-related phenotype in the triple-knockout animals, we isolated genomic DNA from glomeruli of *Phb2*^*pko*^ and *Phb2*^*pko*^*/Insr*^*pko*^*/Igf1r*^*pko*^ mice and performed quantitative PCR experiments specific for the deleted *Phb2* gene. These studies did not detect any differences in *Phb2* gene deletion efficiency between the single- and the triple-knockout mice clearly excluding the possibility of altered recombination efficiency as a basis for the survival advantage (Fig5B). In line with this, *Phb2*^*pko*^*/Insr*^*pko*^*/Igf1r*^*pko*^ mice showed the same mitochondrial structural changes as seen in *Phb2*^*pko*^ mice (Fig5C). Moreover, double-knockout as well as triple-knockout mice developed massive albuminuria (Supplementary Fig S3A and B).

*Insr*^*pko*^*/Igf1r*^*pko*^ showed normal survival for the time analyzed (until 8 weeks after birth) and did not develop proteinuria or renal disease (Supplementary Fig S4A and B) in contrast to previous observations demonstrating renal disease at later time points in life (Welsh *et al*, 2010).

### Loss of PHB2 leads to changes of mitochondrial morphology

**Figure 6:**
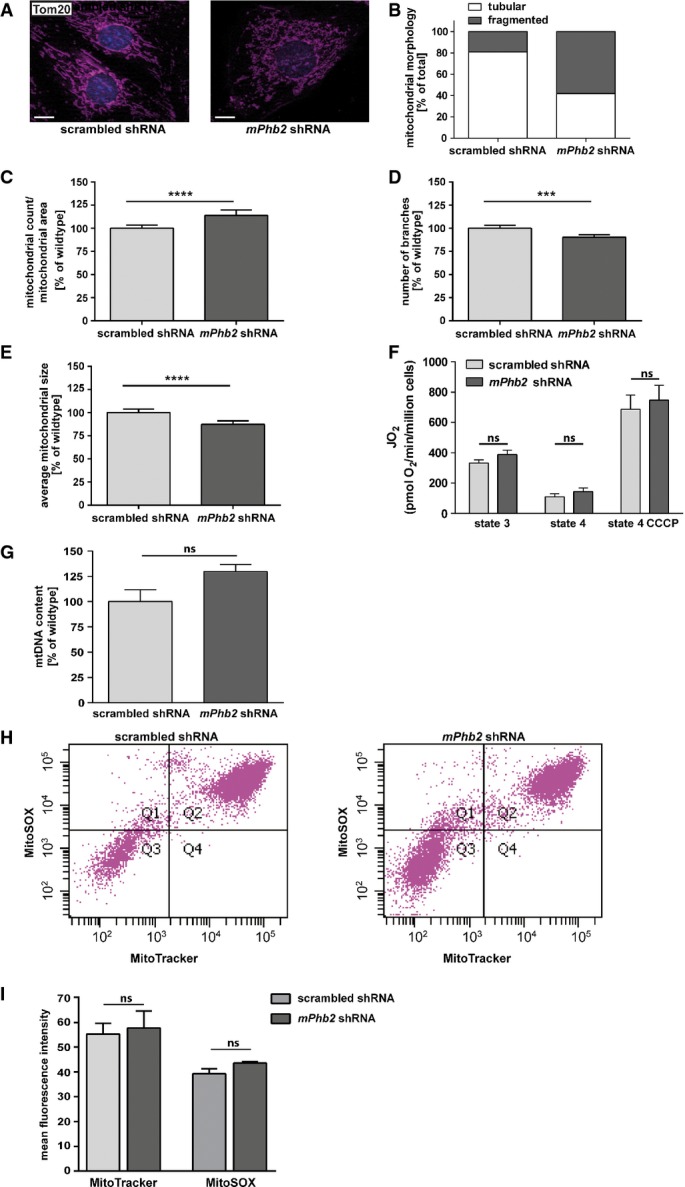
Loss of PHB2 leads to changes in mitochondrial morphology
A Immunofluorescence staining of *mPhb2* shRNA and control podocytes with a Tom20 antibody revealed a disturbed mitochondrial network in *Phb2*-deficient podocytes (scale bar: 10 μm).B Quantification of tubular and fragmented mitochondrial morphology *mPhb2* shRNA versus control podocytes seen in (A) (mitochondrial network of *n* = 100 individual cells of *mPhb2* shRNA podocytes and *n* = 108 individual cells of scrambled shRNA podocytes was assessed).C Morphometric image analysis revealed an increased ratio of mitochondrial count versus mitochondrial area in *Phb2*-deficient podocytes compared to control podocytes (*n* = 75 cell patches for scrambled shRNA, *n *=* *58 cell patches for *mPhb2* shRNA).D Morphometric image analysis showed a decrease in branch numbers in *Phb2*-deficient podocytes compared to control podocytes (*n* = 75 cell patches for scrambled shRNA, *n *=* *58 cell patches for *mPhb2* shRNA).E Morphometric image analysis detected a decrease in mitochondrial size in *Phb2-*deficient podocytes compared to control podocytes (*n* = 75 cell patches for scrambled shRNA, *n *=* *58 cell patches for *mPhb2* shRNA).F Measurements of mitochondrial respiration with complex II substrates (succinate and glycerol-3-phosphate) did not show significant differences between *Phb2-*deficient and control podocytes (*n* = 6).G Quantification of mtDNA as a measure for mitochondrial mass showed similar levels of mtDNA in *Phb2-*deficient podocytes compared to control podocytes (*n* = 3).H FACS analysis of MitoTracker- and MitoSOX-stained *Phb2-*deficient podocytes and control podocytes.I Quantification of mean fluorescence intensity of MitoTracker and MitoSOX by FACS analysis revealed the same mitochondrial mass and the same ROS levels in *Phb2-*deficient podocytes compared to control podocytes (*n *=* *3, *P* = 0.7782 for MitoTracker, *P *=* *0.1088 for MitoSOX).
Data information: In (C–G, I), bars represent mean ± SEM. Student's *t*-test; ****P = 0.0001, ***P =* *0.0002. In (C–E, G), average value for scrambled shRNA podocytes was leveled to 100%.

To better understand the beneficial effect of podocyte-specific insulin signaling deficiency for renal disease and the role of PHB2 on metabolic signaling in podocytes, we next generated a *Phb2* knockdown podocyte cell culture model. As *Phb2* deficiency leads to a reduced cellular proliferation rate impeding the generation of conventional stable cell lines (Merkwirth *et al*, 2008), we utilized a doxycycline-inducible promoter to express short hairpin RNAs directed against *Phb2* mRNA in mouse podocytes (Shankland *et al*, 2007). After doxycycline treatment, decreased mRNA expression of *Phb2* but not *Phb1* was detected (Supplementary Fig S5A). Western blot analysis revealed an interdependence of protein stability of PHB1 and PHB2 and showed reduced protein levels for both PHB1 and PHB2 in *Phb2* knockdown podocytes consistent with previous findings (Artal-Sanz *et al*, 2003; Merkwirth *et al*, 2008) (Supplementary Fig S5B). Knockdown of *Phb2* in podocytes resulted in a disrupted reticular mitochondrial network and the accumulation of fragmented mitochondria (Fig6A and B). The mitochondrial morphology was further analyzed by using morphometric image analysis of the ratio of discrete mitochondrial number to total mitochondrial area as a quantitative measure of mitochondrial length/interconnectivity (Losón *et al*, 2013). These data showed a significant difference between control and *Phb2* knockdown podocytes (Fig6C). Moreover, we detected less branches and smaller mitochondrial sizes after loss of PHB2 (Fig6D and E). However, these structural abnormalities were not accompanied by an impaired oxidative phosphorylation (OXPHOS) system. We did not detect any differences in oxygen consumption rates between *Phb2-*deficient and control podocytes using complex II substrates (succinate and glycerol-3-phosphate) (Fig6F). Similar results were obtained when complex I substrates (pyruvate, glutamate, and malate) were used (unpublished data). Furthermore, there was no evidence for a compensatory upregulation of mitochondrial number as we did not detect differences in total mitochondrial mass by using both, quantification of mtDNA by qPCR (Fig6G) and FACS analysis of podocytes stained with the mitochondrial marker MitoTracker (Fig6H and I). Additionally, we did not detect any change in reactive oxygen species (ROS) in *Phb2*-deficient podocytes (Fig6H and I). These findings are in accordance with previous publications showing an unaltered mitochondrial respiratory function after loss of PHB2 in MEFs (Merkwirth *et al*, 2008). These results indicated that rather a cellular signaling program than a mitochondrial respiratory function defect may account for the severe podocyte dysfunction and cell loss.

### Inhibition of mTOR signaling increases survival of *Phb2*^*pko*^ mice

**Figure 7:**
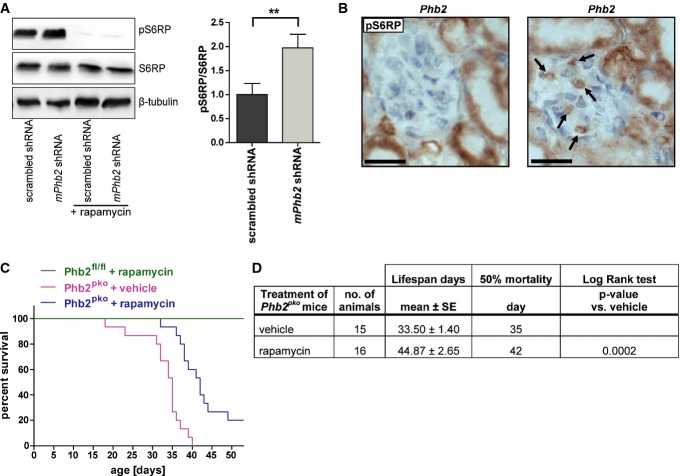
Inhibition of mTOR signaling increases survival of *Phb2*^*pko*^ mice
A *Phb2*-deficient mouse podocytes showed higher levels of phosphorylated S6 ribosomal protein than control podocytes, which could be blocked by treatment with rapamycin (*n* = 5, bars represent mean ± SEM, Student's *t*-test, **P = 0.0036). pS6RP, phosphorylated S6 ribosomal protein; S6RP, S6 ribosomal protein.B Immunohistochemistry for phosphorylated S6 ribosomal protein (pS6RP) on kidney sections revealed increased levels of pS6RP in glomeruli of *Phb2*^*pko*^ compared to *Phb2*^*fl/fl*^ mice (arrows point to cells showing a signal for pS6RP; scale bar: 20 μm).C Kaplan–Meier survival curve revealed that treatment of *Phb2*^*pko*^ mice with rapamycin prolonged survival for several days compared to vehicle-treated *Phb2*^*pko*^ mice (*n* = 11 for *Phb2*^*fl/fl*^ + rapamycin, *n* = 15 for *Phb2*^*pko*^ + vehicle, *n* = 16 for *Phb2*^*pko*^ + rapamycin).D Statistical analysis comparing rapamycin-treated *Phb2*^*pko*^ mice with vehicle-treated *Phb2*^*pko*^ mice.

Hyperactive mTOR in podocytes contributes to kidney disease, and genetic reduction of podocyte-specific mTOR complex 1 (mTORC1) in diabetic animals suppresses the development of diabetic nephropathy (Gödel *et al*, 2011; Inoki *et al*, 2011). Therefore, we next investigated the activity of mTOR signaling in the podocyte cell culture model. Western blot analysis of *Phb2*-deficient podocytes revealed hyperphosphorylation of S6 ribosomal protein (S6RP), a component of the 40S ribosomal subunit, indicative of a hyperactive mTOR signaling pathway (Fig7A) (Magnuson *et al*, 2012). Hyperphosphorylation of S6RP was also observed in *Phb2*-deficient glomeruli *in vivo* (Fig7B). These data indicated that hyperactivity of mTOR signaling may contribute to the detrimental effects of loss of PHB2 in podocytes. And in fact, hyperphosphorylation was partially reversed in the protected *Phb2*^*pko*^*/Insr*^*pko*^*/Igf1r*^*pko*^ triple knockouts, where phosphorylated S6 ribosomal protein was mainly found in non-podocyte cells (Supplementary Fig S6A) and treatment with a dual insulin receptor/IGF-1 receptor inhibitor (BMS 536924, Tocris) decreased phosphorylation of S6RP in *Phb2-*deficient podocytes *in vitro* to the level of control podocytes (Supplementary Fig S6B). These data raise the possibility that mitochondrial dysfunction resulting from *Phb2* deficiency causes hyperactive insulin/mTOR signaling in podocytes. This hypothesis was further supported by experiments in *C. elegans*. Heat-shock treatment of worms results in the nuclear translocation of DAF-16 independent of insulin signaling (Supplementary Fig S7A). Cytoplasmic redistribution, a process known to be controlled by insulin receptor (DAF-2) signaling, was used as an indicator of insulin receptor (DAF-2) signaling in *phb-2*-proficient and *phb-2*-deficient worms (Supplementary Fig S7B–D). Consistent with hyperactive insulin signaling in *phb-2*-deficient animals, loss of PHB-2 accelerated recovery of the DAF-2-dependent cytoplasmic localization of DAF-16 after heat shock (Supplementary Fig S7A–D), further supporting a role for insulin receptor-mediated mTOR hyperactivity resulting from the mitochondrial defects.

Taken together, these data suggested the intriguing possibility that the severe kidney disorder in *Phb2*^*pko*^ mice may be amenable to a therapeutic intervention with mTOR inhibitors. Treatment with the mTOR inhibitor rapamycin abrogated phosphorylation of S6RP in the cell culture model (Fig7A). Daily treatment with rapamycin (3 mg/kg) by intraperitoneal injection beginning at week 2 alleviated the kidney disease and significantly enhanced survival of *Phb2*^*pko*^ animals (Fig7C and D) and reversed the hyperphosphorylation of S6RP (Supplementary Fig S8A), suggesting that at least some of the beneficial effects of insulin and IGF-1 receptor deficiency of podocytes are mediated through preventing mTOR hyperactivity. Interestingly, we did not observe major changes in proteinuria levels which may indicate additional mTOR-independent effects accounting for the proteinuric phenotype (Supplementary Fig S8B).

Since active mTOR inhibits autophagy in podocytes (Inoki & Huber, 2012), we also quantified accumulation of the autophagosome marker LC3 in podocytes *in vitro*. Although a trend to less autophagy was seen in *Phb2*-deficient podocytes compared to control podocytes (Supplementary Fig S9A), this effect was not statistically significant. Even inhibition of autophagosomal degradation by adding chloroquine to the cells did not result in any significant differences in the accumulation of autophagosomes (Supplementary Fig S9A). This is in accordance with our electron microscopy studies where we did not observe a reduced amount of autophagosomes in *Phb2*^*pko*^ mice, suggesting that the mTOR effect may be independent of influencing the level of autophagy.

## Discussion

More than 5% of all human beings worldwide suffer from chronic kidney diseases (CKD) with great impact on quality of life and rising socioeconomic burdens on our societies. Glomerular disorders, with diabetic nephropathy being the leading cause, account for the majority of cases of CKD. In the past several years, accumulating evidence suggested that glomerular podocytes are crucial for the function of the kidney filter and critically involved in the development of glomerulosclerosis and proteinuria (Elger & Kriz, 1998; Brinkkoetter *et al*, 2013). Work within the last decade suggests that both mitochondrial dysfunction and dysregulated insulin signaling contribute to podocyte injury (Coward *et al*, 2005; Welsh *et al*, 2010; Su *et al*, 2013; Zhou *et al*, 2013). Our data indicate that cell intrinsic inhibition of the insulin/IGF-1 signaling system may protect from mitochondria-mediated disease. The link of PHB2 deficiency and altered metabolism and/or insulin signaling had been suggested in yeast and *C. elegans* (Artal-Sanz & Tavernarakis, 2009; Schleit *et al*, 2013). Here, we used a clinically relevant condition, podocyte-driven kidney disease, as a model to show that inhibition of insulin signaling, achieved through cell type specific genetic deletion of insulin receptor alone or in combination with the IGF-1 receptor in podocytes, protects from renal disease, prevents kidney failure, and enhances survival of animals with induced dysfunction of mitochondria. Taken more broadly, these findings provide the intriguing possibility that mitochondrial dysfunction may result in pathological upregulation of insulin/IGF-1 signaling in non-classic insulin-responsive target cells. Whether this is a specific phenomenon for podocytes warrants further investigation.

Staining for the mTORC1 downstream target phosphorylated S6 ribosomal protein revealed enhanced phosphorylation of S6 ribosomal protein in *Phb2*^*pko*^ mice, which was reversed after loss of the insulin and IGF-1 receptor. This raised the intriguing hypothesis that the beneficial effect of the loss of insulin signaling in *Phb2*^*pko*^ mice depends on less active mTORC1. In line with this, we also observed that inhibition of mTOR kinase activity with rapamycin protects from disease and robustly increases survival. Our data perfectly complement with recent data provided by Kaeberlein and colleagues who demonstrated a convincing beneficial effect of mTOR inhibition in a rare mitochondrial disease with mainly neurological symptoms (Johnson *et al*, 2013). Moreover, a recent study in zebrafish linked mutations in electron transfer proteins, which are associated with severe inherited mitochondrial disorders due to defects in β-oxidation, to increased signaling via mTOR (Kim *et al*, 2013), further emphasizing the connection between mitochondria and metabolic signaling. Here, we show that defects in the mitochondrial fusion machinery, which become evident after loss of PHB2, lead to hyperactivation of the insulin/IGF-1 signaling pathway and subsequently mTOR. There is an increasing body of literature that supports the hypothesis that malfunctioning mitochondria may cause diseases by sending pathogenic signals and altering the cellular metabolism rather than by inhibiting respiratory chain activity and lowering energy levels (reviewed in (Raimundo, 2014)).

Our findings may have important implications as mitochondrial dysfunction, impaired metabolism, and alterations in the production of reactive oxygen species have been implicated in renal aging and a large variety of kidney disease, most of them without any available treatment.

## Materials and Methods

### Antibodies

The antibodies used in this study are listed in Table1.

**Table 1:** Antibodies used in this study

Name	Company	Catalog no.	Species	Dilution
Anti-cleaved caspase-3	Cell Signaling	9661	Rabbit	1:200
Anti-LC3	MBL	PM036	Rabbit	1:500 (WB)
Anti-PHB1	BioLegend	603101	Rabbit	1:1,000
Anti-PHB2	BioLegend	611802	Rabbit	1:1,000
Anti-podocin	Sigma	P0372	Rabbit	1:1,000
Anti-phospho-S6 ribosomal protein	Cell Signaling	4858	Rabbit	1:2,000 (WB); 1:100 (IF)
Anti-S6 ribosomal protein	Cell Signaling	2217	Rabbit	1:2,000
Anti-Tom20 (FL-145)	Santa Cruz	sc-11415	Rabbit	1:200
Anti-WT-1 (C-19)	Santa Cruz	sc-192	Rabbit	1:1,000

### Mouse models

Mice in which exons 3 and 4 of the *Phb2* gene are flanked by two loxP sequences (*Phb2 flox/flox* mice; Merkwirth *et al*, 2008) were mated to either NPHS2.Cre mice (Moeller *et al*, 2003) or tamoxifen-inducible podocin-iCreER(T2) (Wang *et al*, 2010) to generate podocyte-specific *Phb2* knockout mice *Phb2*^*fl/fl*^;NPHS2.Cre (*Phb2*^*pko*^) or tamoxifen-inducible podocyte-specific *Phb2* knockout mice *Phb2*^*fl/fl*^; podocin-iCreER(T2) [podocin-iCreER(T2) *Phb2*^*pko*^]. We included mice from both genders carrying the conventional NPHS2.cre transgene while only 8-week-old male animals were exposed to a tamoxifen-enriched diet (400 mg/kg; TD.55125; Harlan Laboratories, Indianapolis, IN, USA) *ad libitum* for 6 weeks to exclude any potential side effects in female animals. Based on a daily intake of 5 g of food per mouse, this corresponded to an oral dose of 2 mg/day tamoxifen per mouse.

To generate double- and triple-knockout mice, first, *Phb2 flox/flox* mice were mated to *Igf1r flox/flox* and/or *Insr flox/flox* mice. Second, these mice were mated to podocyte-specific Cre mice (*NPHS2.cre* mice) (Moeller *et al*, 2003) to obtain mice with a podocyte-specific deficiency of either *Phb2* and *Igf1r* (*Phb2*^*pko*^*/Igf1r*^*pko*^ mice) or *Phb2* and *Insr* (*Phb2*^*pko*^*/Insr*^*pko*^ mice) or *Phb2* and *Insr* and *Igf1r* (*Phb2*^*pko*^*/Insr*^*pko*^*/Igf1r*^*pko*^ mice).

All animals were backcrossed onto the C57BL/6 background for at least 10 generations. Mice were housed according to the standardized specific pathogen-free conditions in the University of Cologne animal facility. The Animal Care Committee of the University of Cologne reviewed and approved the experimental protocol. The mice were sacrificed once they lost 15–20% of their maximal body weight following federal animal care regulations. Urine was analyzed with Coomassie gel stains, ELISA (mouse albumin ELISA kit; Bethyl Labs, Montgomery, TX, USA), and urine creatinine assay (Cayman Chemical, Ann Arbor, MI, USA). Blood samples were collected in the fifth week of life for quantification of serum urea and creatinine by the Institute for Clinical Chemistry of the University Hospital of Cologne, Germany. Renal tissue was embedded in OCT compound (Sakura, Torrance, CA, USA) and frozen at −70°C or fixed in 4% neutral buffered formalin for immunostaining.

For rapamycin injections, the protocol published by Zeng *et al* (2008) was used with some modifications. Animals received daily i.p. injections of either rapamycin solution (LC Laboratories, Woburn, MA, USA) or vehicle solution [5% (v/v) ethanol, 5% (v/v) Tween® 80, 5% (v/v) PEG400] starting 2 weeks after birth until they were sacrificed because of weight loss or sickness. *Phb2*^*pko*^ and control mice were injected with either 3 μg rapamycin solution per g body weight or the same volume of vehicle solution (DMSO). Rapamycin solution was always freshly prepared prior to injection. Animals were randomly assigned to different treatment groups independently of genotyping results.

The sample size for the animal studies was based on ANOVA calculations according to the variable-criteria sequential stopping rule (SSR). All analyses and quantifications were performed in a blinded fashion, and no animals were excluded from the evaluation or the statistics.

### Immunohistochemistry

Indirect immunoperoxidase staining was performed on formalin-fixed tissue. Briefly, 4-μm-thin tissue sections were deparaffinized in Xylene (VWR, Darmstadt, Germany) and rehydrated in graded ethanol. Endogenous peroxidase activity was blocked with 3% hydrogen peroxidase (Fischar, Saarbruecken, Germany). Sections were incubated overnight at 4°C with primary antibodies diluted in 1% BSA/TBS. The sections were washed repeatedly in TBS before incubation with biotinylated mouse anti-rabbit secondary antibody (Jackson Immunoresearch, West Grove, PA, USA) diluted in 1% BSA/TBS for 1 h at room temperature. The ABC kit (Vector, Burlingame, CA, USA) was used for signal amplification, and 3,3′-diaminobenzamidine (Sigma-Aldrich, St Louis, MO, USA) was used as a chromogen. Slides were counterstained with hematoxylin (Sigma-Aldrich), dehydrated, and covered with Histomount (National Diagnostics, Atlanta, GA, USA). Periodic acid Schiff (PAS) staining was performed for the assessment of glomerulosclerosis. Images were acquired with an Axiovert 200 M microscope/EC Plan-Neofluar ×40/1.3 oil immersion or C-Apo ×63/1.20 water immersion objective equipped with a charge-coupled-device camera (all from Carl Zeiss MicroImaging GmbH, Jena, Germany). Images were further processed using ImageJ/Fiji software version 1.46 (NIH, Bethesda, MD, USA).

### Immunofluorescence

Fresh cut frozen sections were fixed with 4% PFA before overnight incubation at 4°C with anti-phospho-S6 ribosomal protein (Ser235/236) from Cell Signaling, Danvers, MA, diluted in 5% NDS/PBS+Triton X-100. The sections were washed repeatedly with PBS before incubation with fluorescently labeled anti-rabbit secondary antibody (Jackson Immunoresearch) diluted in PBS for 1 h at room temperature. Anti-Podocin from Sigma-Aldrich was directly labeled with a fluorescent dye according to the manufacturer's instructions (Zenon® Rabbit IgG Labeling Kit; Life Technologies, Carlsbad, CA, USA). Sections were mounted with ProLong® Gold antifade reagent (Life Technologies).

Cells were fixed with 4% PFA before overnight incubation at 4°C with the primary antibody diluted in 5% NDS/PBS+Triton X-100. The sections were washed repeatedly with PBS before incubation with the fluorescently labeled secondary antibody (Jackson Immunoresearch) diluted in PBS for 1 h at room temperature. Mounting was done with ProLong® Gold antifade reagent (Life Technologies). Images were acquired with an Axiovert 200 M microscope/EC Plan-Neofluar ×40/1.3 oil immersion or C-Apo ×63/1.20 water immersion objective equipped with a charge-coupled-device camera (all from Carl Zeiss MicroImaging GmbH) or an LSM 710/Axiobserver Z1 confocal microscope ×63/1.4 oil immersion objective operated by ZEN 2009 software. Images were further processed and analyzed using ImageJ/Fiji software version 1.46 (NIH; Schindelin *et al*, 2012). For morphometric analysis, images were processed using median and Mexican hat filtering (http://rsb.info.nih.gov/ij/plugins/mexican-hat/index.html). Mitochondria were then thresholded and binarized for subsequent analysis. Branching on skeletonized binary images was measured using the Analyse Skeleton plugin (Arganda-Carreras *et al*, 2010).

### Electron microscopy

Mice were perfused with electron microscopy fixation buffer (4% paraformaldehyde and 2% glutaraldehyde in 0.1 M sodium cacodylate, pH 7.4) and the kidneys post-fixed in the same buffer for two weeks at 4°C. Samples were osmicated with 1% OsO4 in 0.1 M cacodylate and dehydrated in a graduated ethanol series. Infiltration with Epon and flat embedding was performed according to standard procedures. Thin (30 nm) cross sections were taken on an Ultracut UCT ultramicrotome (Reichert, Heidelberg, Germany). The sections were stained with 1% aqueous uranylic acetate and lead citrate and examined with a Zeiss EM 902 electron microscope (LEO, Oberkochen, Germany). Images were further processed with Adobe Photoshop CS4 version 11.0.0.0. (Adobe Systems, San Jose, CA, USA).

### Cell culture

Conditionally immortalized podocytes were generated as previously described (Shankland *et al*, 2007) and cultured in RPMI media supplemented with 10% FBS and IFNγ. Mycoplasm contamination was excluded by PCR testing. Short hairpin RNAs (shRNAs) against PHB2 were designed based on the prediction of a publicly available prediction program (RNAi Designer; Invitrogen, Carlsbad, CA, USA). shRNAs were selected for efficient knockdown by using a Dual-luciferase® reporter assay (Promega, Madison, WI, USA). Podocytes containing either a doxycycline-inducible scrambled shRNA (5′-TGCTGAAATGTACTGCGCGTGGAGACGTTTTGGCCACTGACTGACGTCTCCACGCAGTACATTT-3′) or two Phb2 shRNAs (5′-TGCTGAGCTAAGTCCTTCAAGTTCTGGTTTTGGCCACTGACTGACCAGAACTTAGGACTTAGCT-3′ and 5′-TGCTGTAACAATGGACGGCAGCACTCGTTTTGGCCACTGACTGACGAGTGCTGGTCCATTGTTA-3′) were generated by means of the T-Rex™ System by Invitrogen (Invitrogen). If not indicated otherwise, differentiation of podocytes was induced by culturing the cells at 37°C on Primaria plastic plates (BD Biosciences, San Jose, CA, USA) in the absence of IFNγ. After 10 days of differentiation, 2 μg/ml doxycycline was added to the medium and exchanged every 24 h. Experiments were performed after 96 h of doxycycline treatment. In our inhibition experiments, cells were treated either for 2 h with 10 ng/ml rapamycin (LC Laboratories) or for 24 h with 10 μM BMS 53692 (Tocris).

Autophagy experiments were carried out with undifferentiated podocytes at 33°C after treatment with 2 μg/ml for 96 h. Undifferentiated podocytes were used for this experiment because LC3 levels were below the detection limit in cultured differentiated podocytes. In this experiment, cells were treated with 10 ng/ml rapamycin for 24 h and 25 μM chloroquine for 2 h.

### Statistical analysis

All results are expressed as means ± SEM. Statistical significance was evaluated using GraphPad Prism version 4.00c for Macintosh and OASIS (Online Application for the Survival Analysis of Lifespan Assays) (Yang *et al*, 2011). Unpaired Student's *t*-test was applied in most experiments. A Log rank test (Bewick *et al*, 2004) was performed for survival curves, and a chi-square test was applied for the subcellular localization of DAF-16::GFP. A *P*-value < 0.05 was considered significant.

For more detailed Materials and Methods, see Supplementary Information.

## Supporting Information

Supplementary Information

Review Process File

## References

[b1] Arganda-Carreras I, Fernández-González R, Muñoz-Barrutia A, Ortiz-De-Solorzano C (2010). 3D reconstruction of histological sections: application to mammary gland tissue. Microsc Res Tech.

[b2] Artal-Sanz M, Tsang WY, Willems EM, Grivell LA, Lemire BD, van der Spek H, Nijtmans LGJ, Sanz MA (2003). The mitochondrial prohibitin complex is essential for embryonic viability and germline function in *Caenorhabditis elegans*. J Biol Chem.

[b3] Artal-Sanz M, Tavernarakis N (2009). Prohibitin couples diapause signalling to mitochondrial metabolism during ageing in *C. elegans*. Nature.

[b4] Back JW, Sanz MA, De Jong L, De Koning LJ, Nijtmans LGJ, De Koster CG, Grivell LA, Van Der Spek H, Muijsers AO (2002). A structure for the yeast prohibitin complex: structure prediction and evidence from chemical crosslinking and mass spectrometry. Protein Sci.

[b5] Baris OR, Klose A, Kloepper JE, Weiland D, Neuhaus JFG, Schauen M, Wille A, Müller A, Merkwirth C, Langer T (2011). The mitochondrial electron transport chain is dispensable for proliferation and differentiation of epidermal progenitor cells. Stem Cells.

[b6] Bewick V, Cheek L, Ball J (2004). Statistics review 12: survival analysis. Crit Care.

[b9] Bratic A, Larsson N-G (2013). The role of mitochondria in aging. J Clin Invest.

[b11] Brinkkoetter PT, Ising C, Benzing T (2013). The role of the podocyte in albumin filtration. Nat Rev Nephrol.

[b12] Coward RJM, Welsh GI, Yang J, Tasman C, Lennon R, Koziell A, Satchell S, Holman GD, Kerjaschki D, Tavaré JM (2005). The human glomerular podocyte is a novel target for insulin action. Diabetes.

[b13] Elger M, Kriz W (1998). Podocytes and the development of segmental glomerulosclerosis. Nephrol Dial Transplant.

[b15] Gödel M, Hartleben B, Herbach N, Liu S, Zschiedrich S, Lu S, Debreczeni-Mór A, Lindenmeyer MT, Rastaldi M-P, Hartleben G (2011). Role of mTOR in podocyte function and diabetic nephropathy in humans and mice. J Clin Invest.

[b17] Imasawa T, Rossignol R (2013). Podocyte energy metabolism and glomerular diseases. Int J Biochem Cell Biol.

[b18] Inoki K, Mori H, Wang J, Suzuki T, Hong S, Yoshida S, Blattner SM, Ikenoue T, Rüegg MA, Hall MN (2011). mTORC1 activation in podocytes is a critical step in the development of diabetic nephropathy in mice. J Clin Invest.

[b19] Inoki K, Huber TB (2012). Mammalian target of rapamycin signaling in the podocyte. Curr Opin Nephrol Hypertens.

[b20] Johnson SC, Yanos ME, Kayser E-B, Quintana A, Sangesland M, Castanza A, Uhde L, Hui J, Wall VZ, Gagnidze A (2013). mTOR inhibition alleviates mitochondrial disease in a mouse model of Leigh syndrome. Science.

[b22] Kasashima K, Sumitani M, Satoh M, Endo H (2008). Human prohibitin 1 maintains the organization and stability of the mitochondrial nucleoids. Exp Cell Res.

[b23] Kim S-H, Scott SA, Bennett MJ, Carson RP, Fessel J, Brown HA, Ess KC (2013). Multi-organ abnormalities and mTORC1 activation in zebrafish model of multiple acyl-CoA dehydrogenase deficiency. PLoS Genet.

[b24] Losón OC, Song Z, Chen H, Chan DC (2013). Fis1, Mff, MiD49, and MiD51 mediate Drp1 recruitment in mitochondrial fission. Mol Biol Cell.

[b25] Magnuson B, Ekim B, Fingar DC (2012). Regulation and function of ribosomal protein S6 kinase (S6K) within mTOR signalling networks. Biochem J.

[b26] Merkwirth C, Dargazanli S, Tatsuta T, Geimer S, Löwer B, Wunderlich FT, von Kleist-Retzow J-C, Waisman A, Westermann B, Langer T (2008). Prohibitins control cell proliferation and apoptosis by regulating OPA1-dependent cristae morphogenesis in mitochondria. Genes Dev.

[b27] Merkwirth C, Langer T (2009). Prohibitin function within mitochondria: essential roles for cell proliferation and cristae morphogenesis. Biochim Biophys Acta.

[b28] Merkwirth C, Martinelli P, Korwitz A, Morbin M, Brönneke HS, Jordan SD, Rugarli EI, Langer T (2012). Loss of prohibitin membrane scaffolds impairs mitochondrial architecture and leads to tau hyperphosphorylation and neurodegeneration. PLoS Genet.

[b29] Moeller MJ, Sanden SK, Soofi A, Wiggins RC, Holzman LB (2003). Podocyte-specific expression of cre recombinase in transgenic mice. Genesis.

[b31] Nijtmans LG, de Jong L, Artal Sanz M, Coates PJ, Berden JA, Back JW, Muijsers AO, van der Spek H, Grivell LA (2000). Prohibitins act as a membrane-bound chaperone for the stabilization of mitochondrial proteins. EMBO J.

[b32] Nunnari J, Suomalainen A (2012). Mitochondria: in sickness and in health. Cell.

[b33] Osman C, Haag M, Potting C, Rodenfels J, Dip PV, Wieland FT, Brügger B, Westermann B, Langer T (2009a). The genetic interactome of prohibitins: coordinated control of cardiolipin and phosphatidylethanolamine by conserved regulators in mitochondria. J Cell Biol.

[b34] Osman C, Merkwirth C, Langer T (2009b). Prohibitins and the functional compartmentalization of mitochondrial membranes. J Cell Sci.

[b35] Park S-E, Xu J, Frolova A, Liao L, O'Malley BW, Katzenellenbogen BS (2005). Genetic deletion of the repressor of estrogen receptor activity (REA) enhances the response to estrogen in target tissues in vivo. Mol Cell Biol.

[b37] Raimundo N (2014). Mitochondrial pathology: stress signals from the energy factory. Trends Mol Med.

[b38] Schindelin J, Arganda-Carreras I, Frise E, Kaynig V, Longair M, Pietzsch T, Preibisch S, Rueden C, Saalfeld S, Schmid B (2012). Fiji: an open-source platform for biological-image analysis. Nat Methods.

[b39] Schleit J, Johnson SC, Bennett CF, Simko M, Trongtham N, Castanza A, Hsieh EJ, Moller RM, Wasko BM, Delaney JR (2013). Molecular mechanisms underlying genotype-dependent responses to dietary restriction. Aging Cell.

[b40] Shankland SJ, Pippin JW, Reiser J, Mundel P (2007). Podocytes in culture: past, present, and future. Kidney Int.

[b41] Steglich G, Neupert W, Langer T (1999). Prohibitins regulate membrane protein degradation by the m-AAA protease in mitochondria. Mol Cell Biol.

[b42] Su M, Dhoopun A-R, Yuan Y, Huang S, Zhu C, Ding G, Liu B, Yang T, Zhang A (2013). Mitochondrial dysfunction is an early event in aldosterone-induced podocyte injury. Am J Physiol Renal Physiol.

[b43] Tatsuta T, Model K, Langer T (2005). Formation of membrane-bound ring complexes by prohibitins in mitochondria. Mol Biol Cell.

[b44] Wang J, Wang Y, Long J, Chang BHJ, Wilson MH, Overbeek P, Danesh FR (2010). Tamoxifen-inducible podocyte-specific iCre recombinase transgenic mouse provides a simple approach for modulation of podocytes in vivo. Genesis.

[b45] Welsh GI, Hale LJ, Eremina V, Jeansson M, Maezawa Y, Lennon R, Pons DA, Owen RJ, Satchell SC, Miles MJ (2010). Insulin signaling to the glomerular podocyte is critical for normal kidney function. Cell Metab.

[b46] Wu Q, Wu S (2012). Lipid rafts association and anti-apoptotic function of prohibitin in ultraviolet B light-irradiated HaCaT keratinocytes. Exp Dermatol.

[b47] Yang J-S, Nam H-J, Seo M, Han SK, Choi Y, Nam HG, Lee S-J, Kim S (2011). OASIS: online application for the survival analysis of lifespan assays performed in aging research. PLoS One.

[b48] Zeng L-H, Xu L, Gutmann DH, Wong M (2008). Rapamycin prevents epilepsy in a mouse model of tuberous sclerosis complex. Ann Neurol.

[b49] Zhou Y, Bian X, Fang L, He W, Dai C, Yang J (2013). Aristolochic Acid causes albuminuria by promoting mitochondrial DNA damage and dysfunction in podocyte. PLoS One.

