# Supplementary figures and images for: Inhibition of insulin/IGF-1 receptor signaling protects from mitochondria-mediated kidney failure
Source: EMBO Mol Med. 2015 Feb 2;7(3):275–87. doi: 10.15252/emmm.201404916 (PMC4364945; doi:10.15252/emmm.201404916)

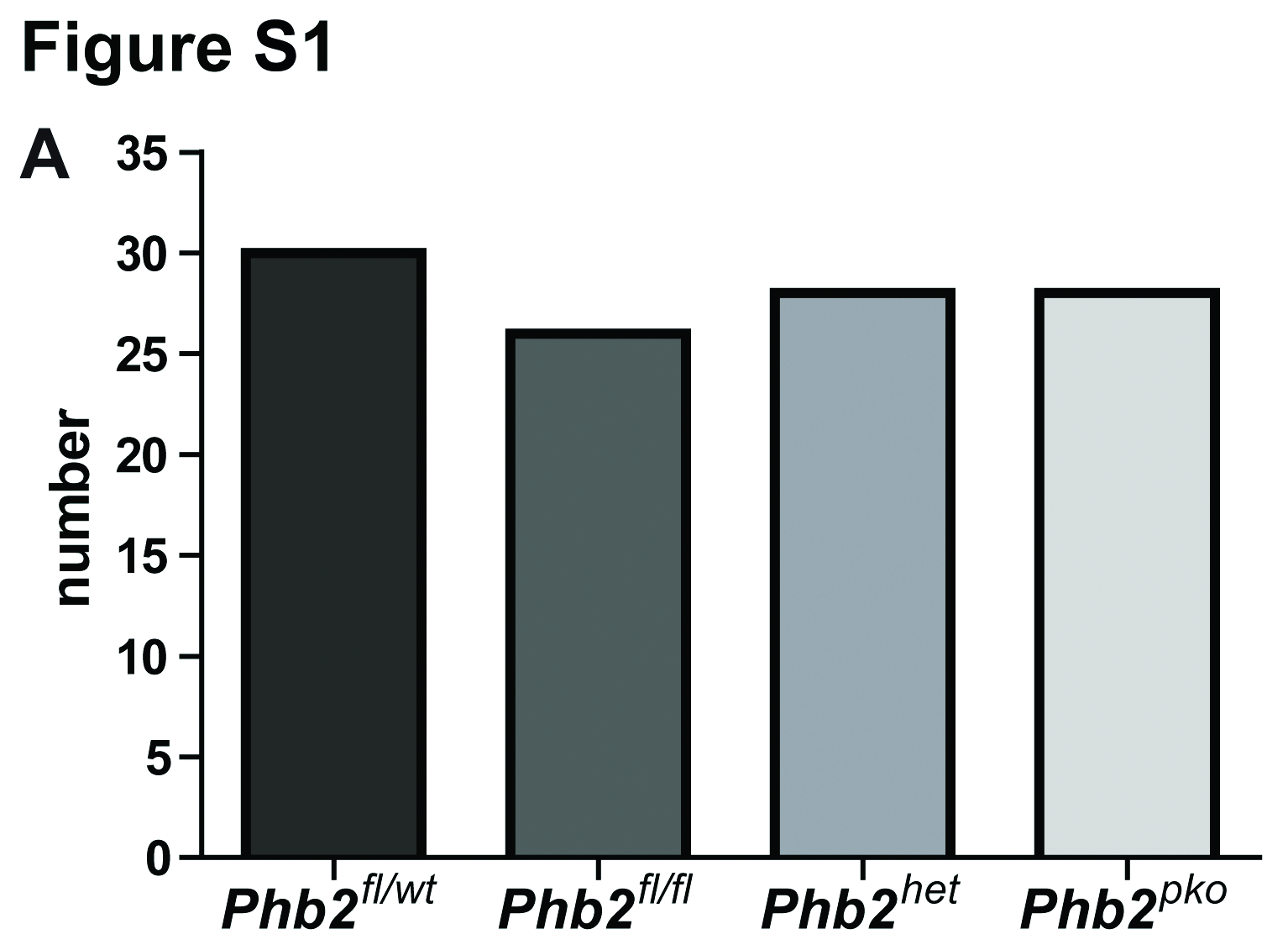

Supplement: Supplementary file 1 [file emmm0007-0275-sd1.tif]

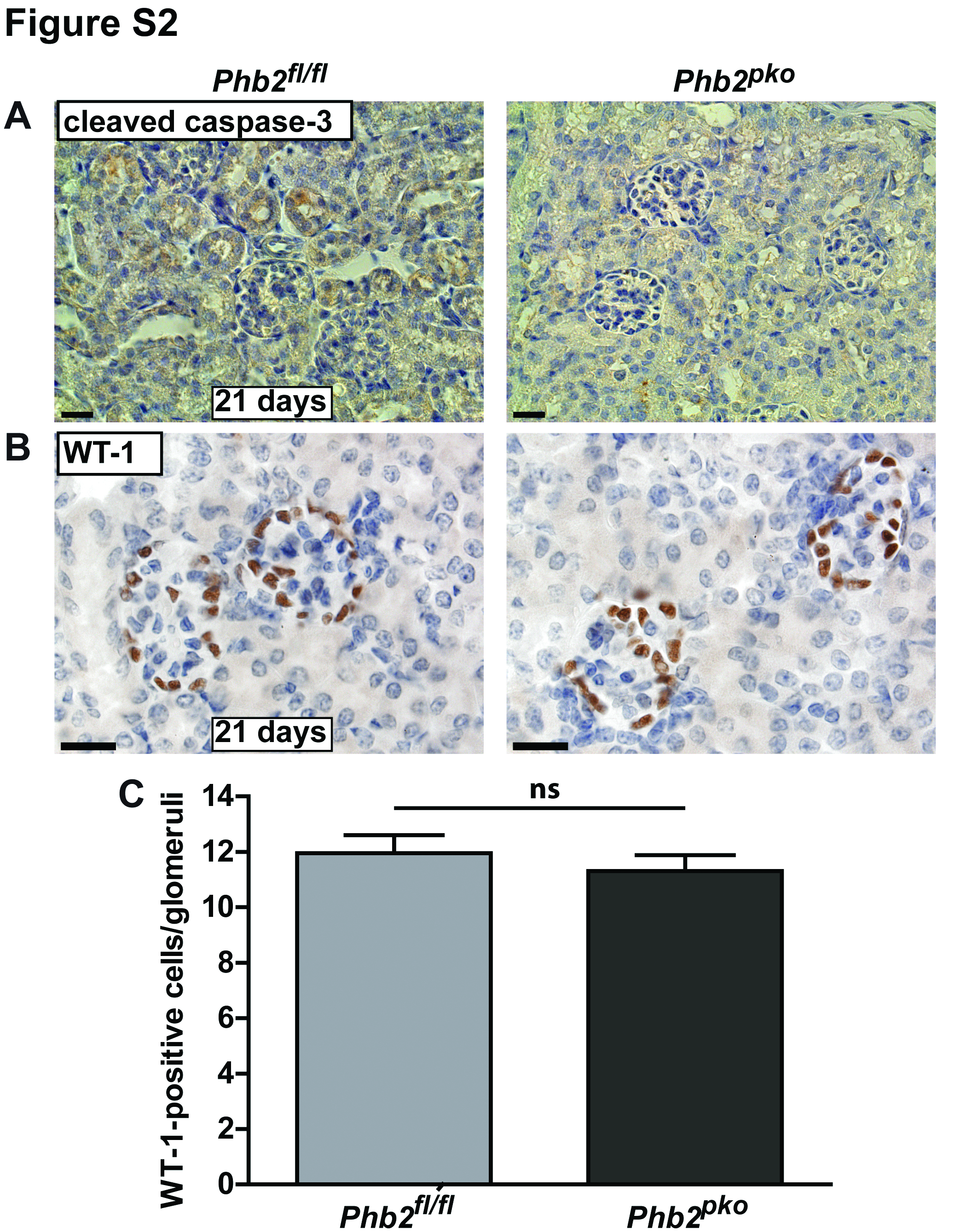

Supplement: Supplementary file 2 [file emmm0007-0275-sd2.tif]

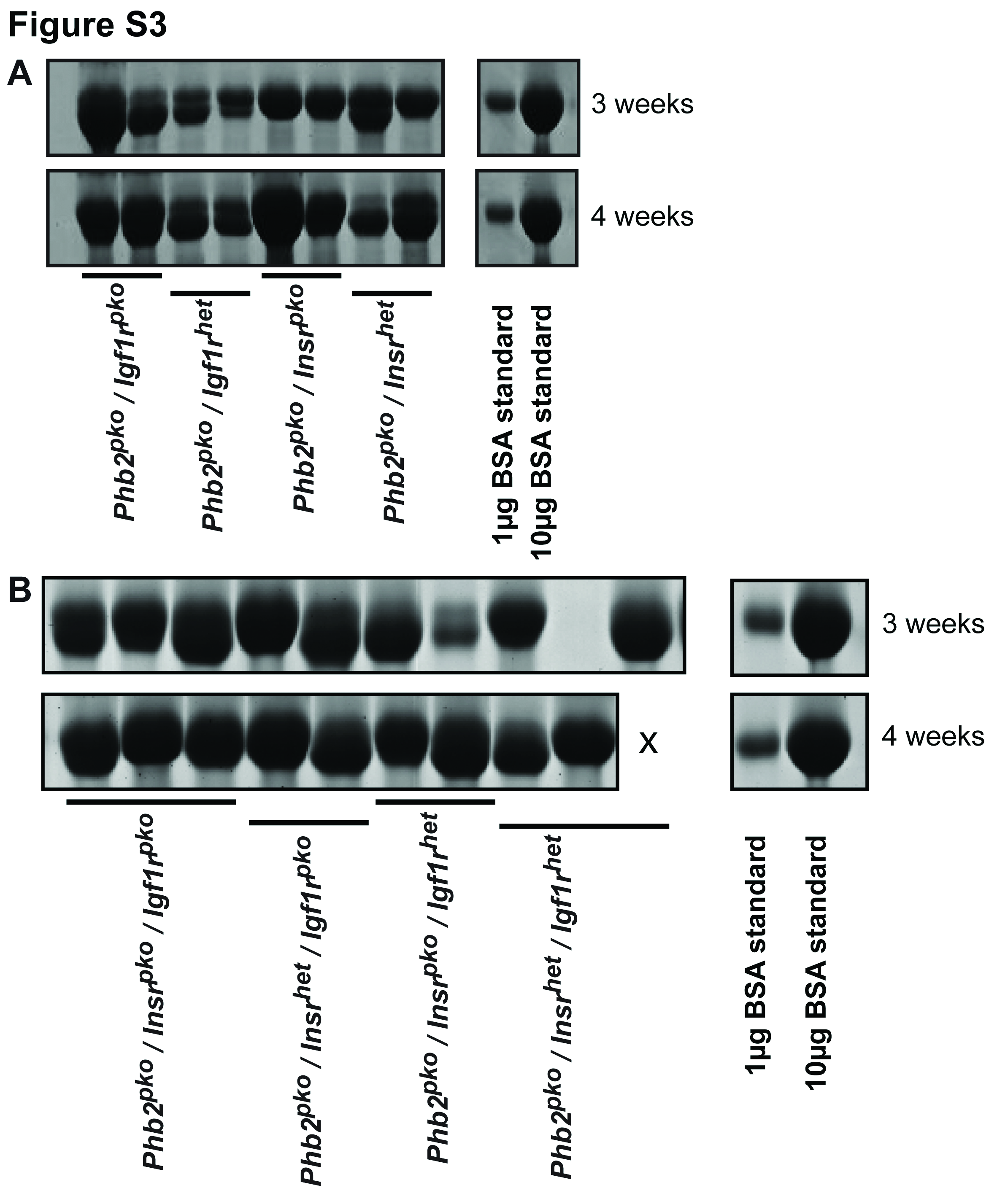

Supplement: Supplementary file 3 [file emmm0007-0275-sd3.tif]

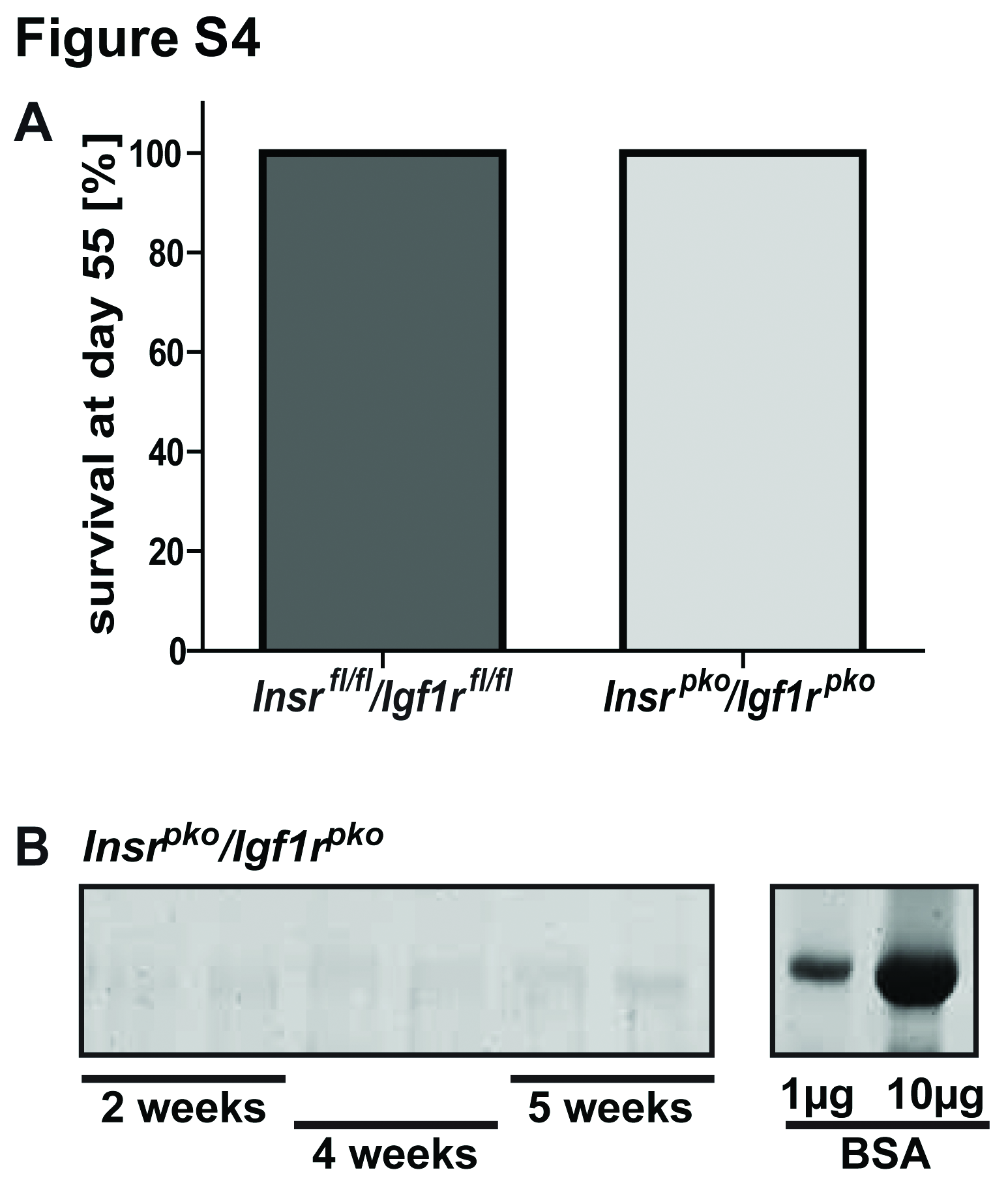

Supplement: Supplementary file 4 [file emmm0007-0275-sd4.tif]

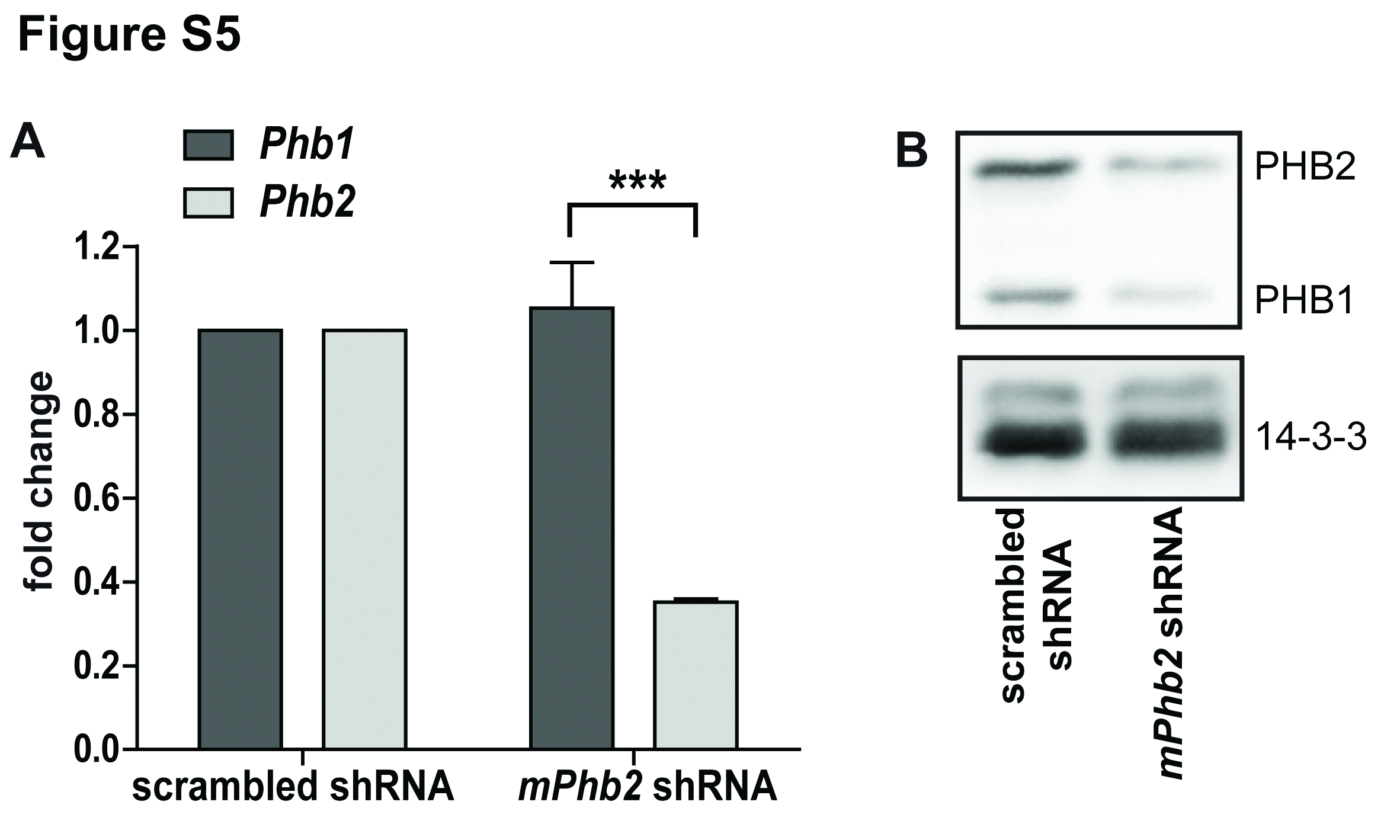

Supplement: Supplementary file 5 [file emmm0007-0275-sd5.tif]

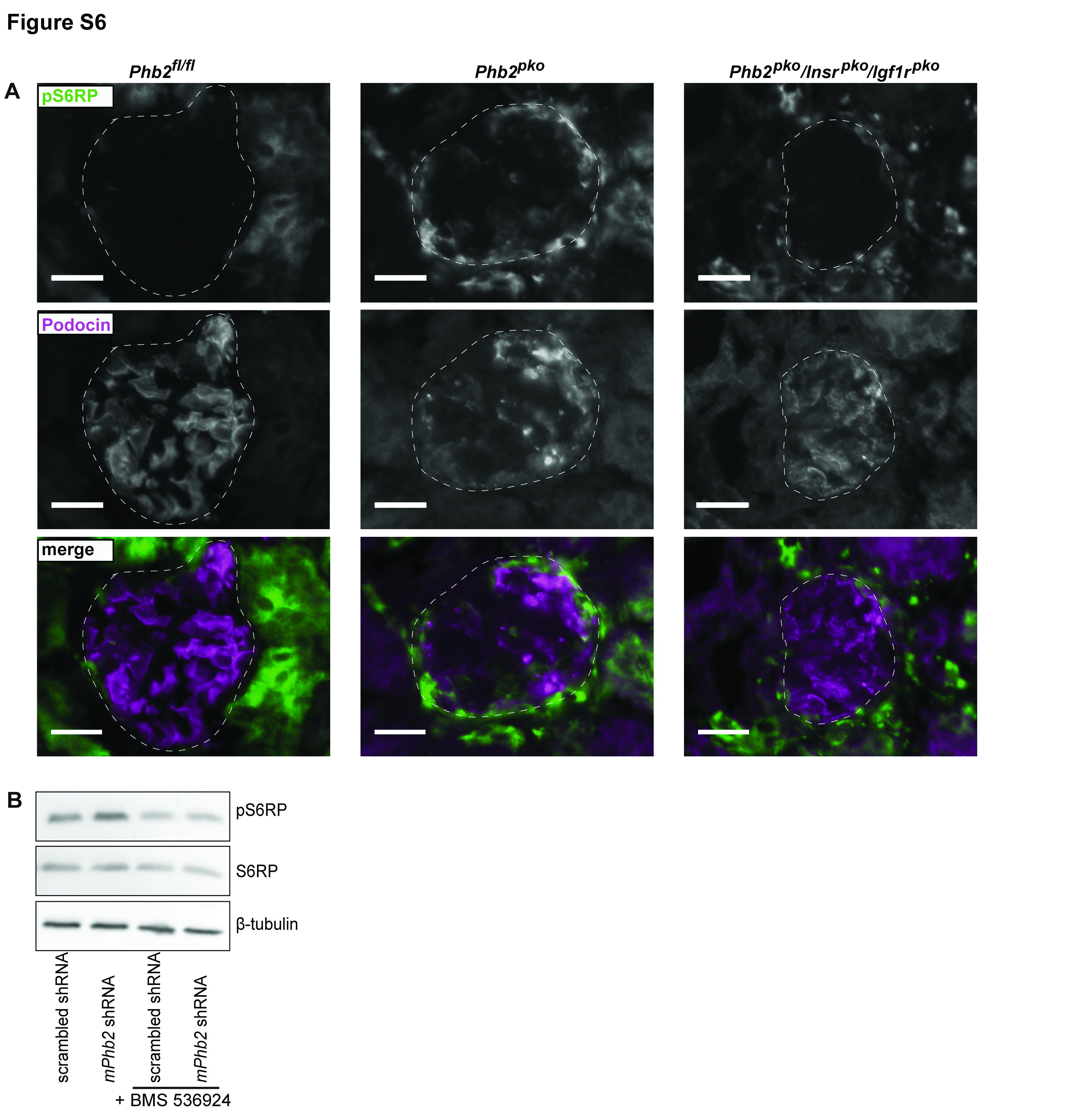

Supplement: Supplementary file 6 [file emmm0007-0275-sd6.tif]

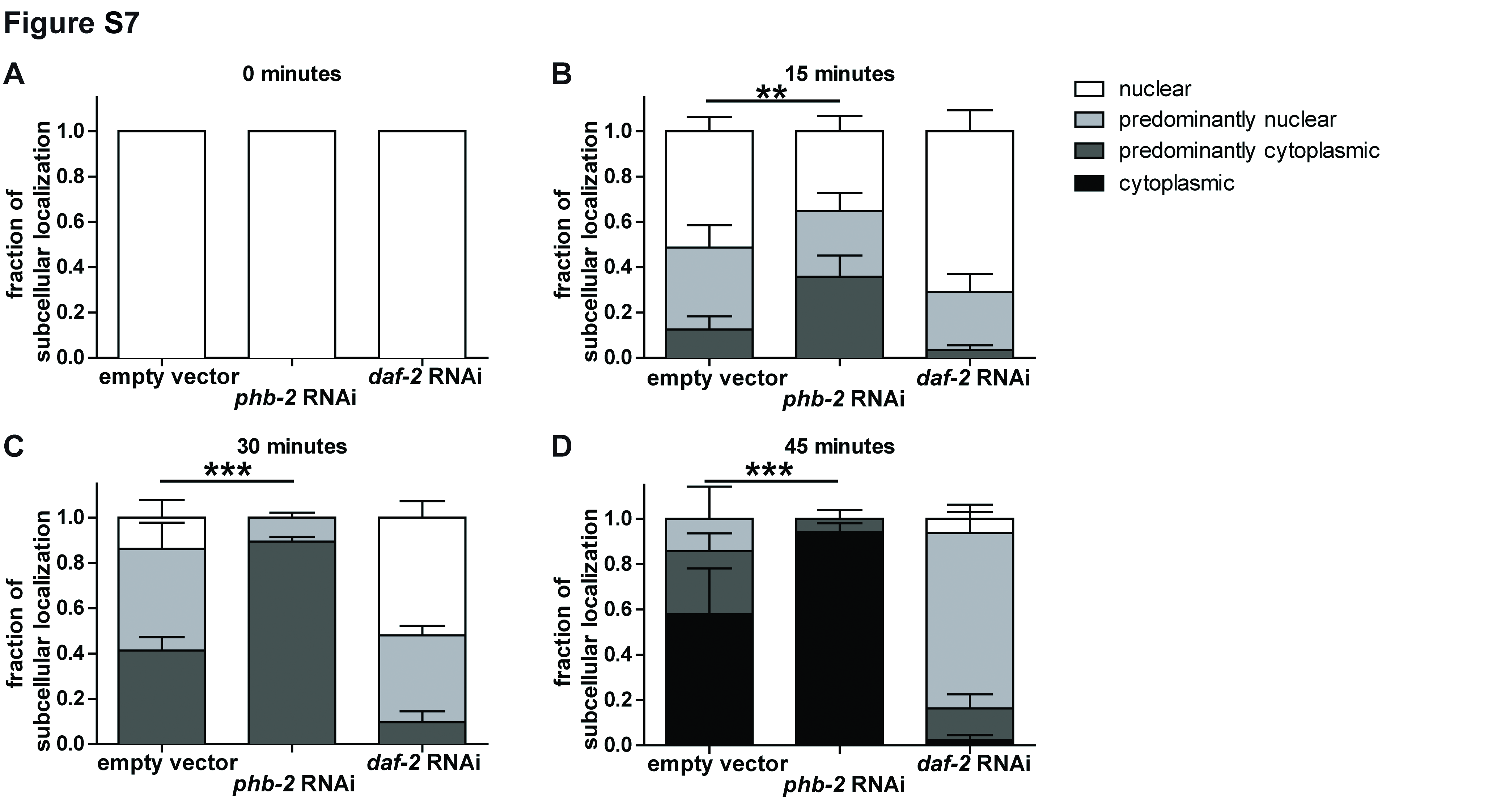

Supplement: Supplementary file 7 [file emmm0007-0275-sd7.tif]

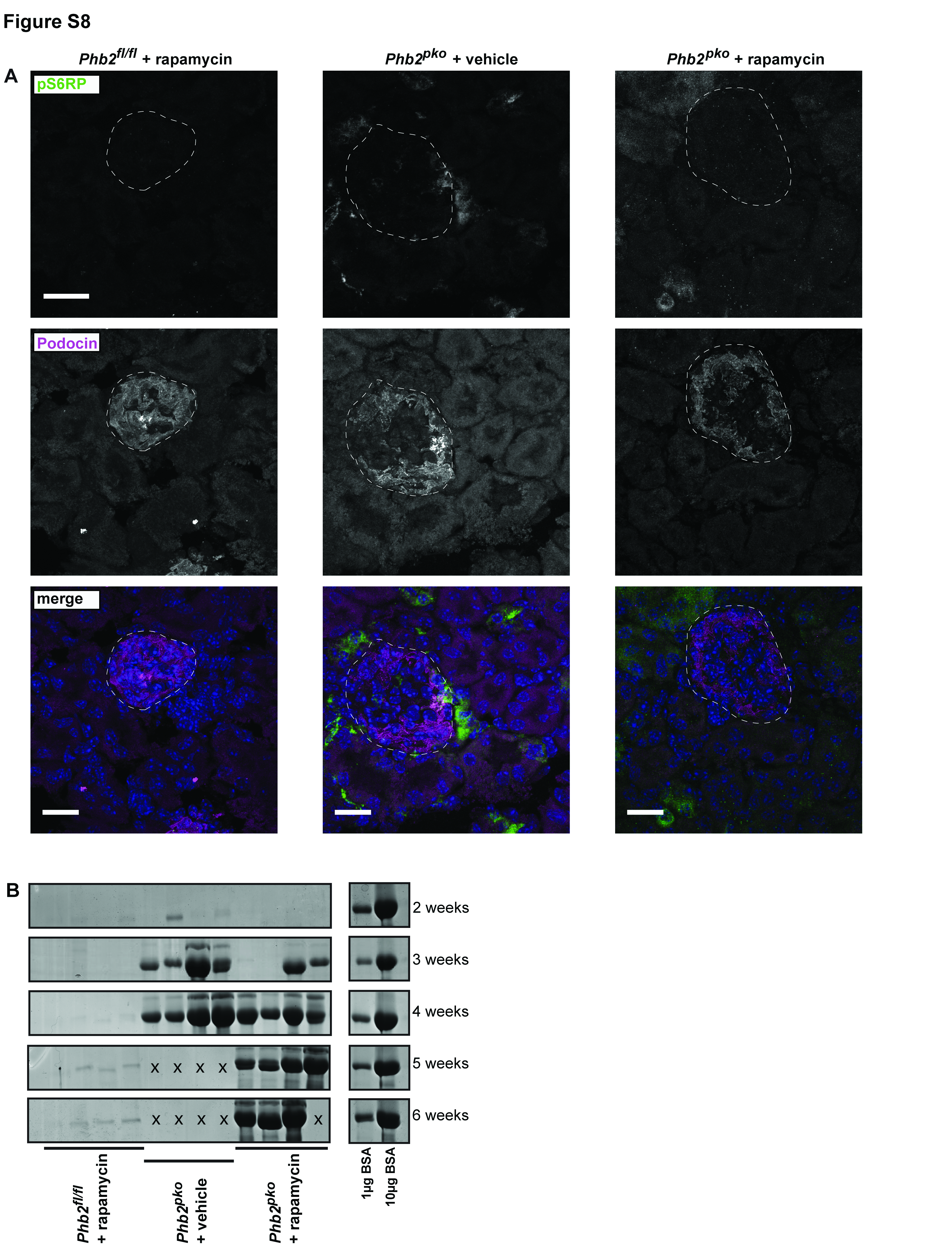

Supplement: Supplementary file 8 [file emmm0007-0275-sd8.tif]

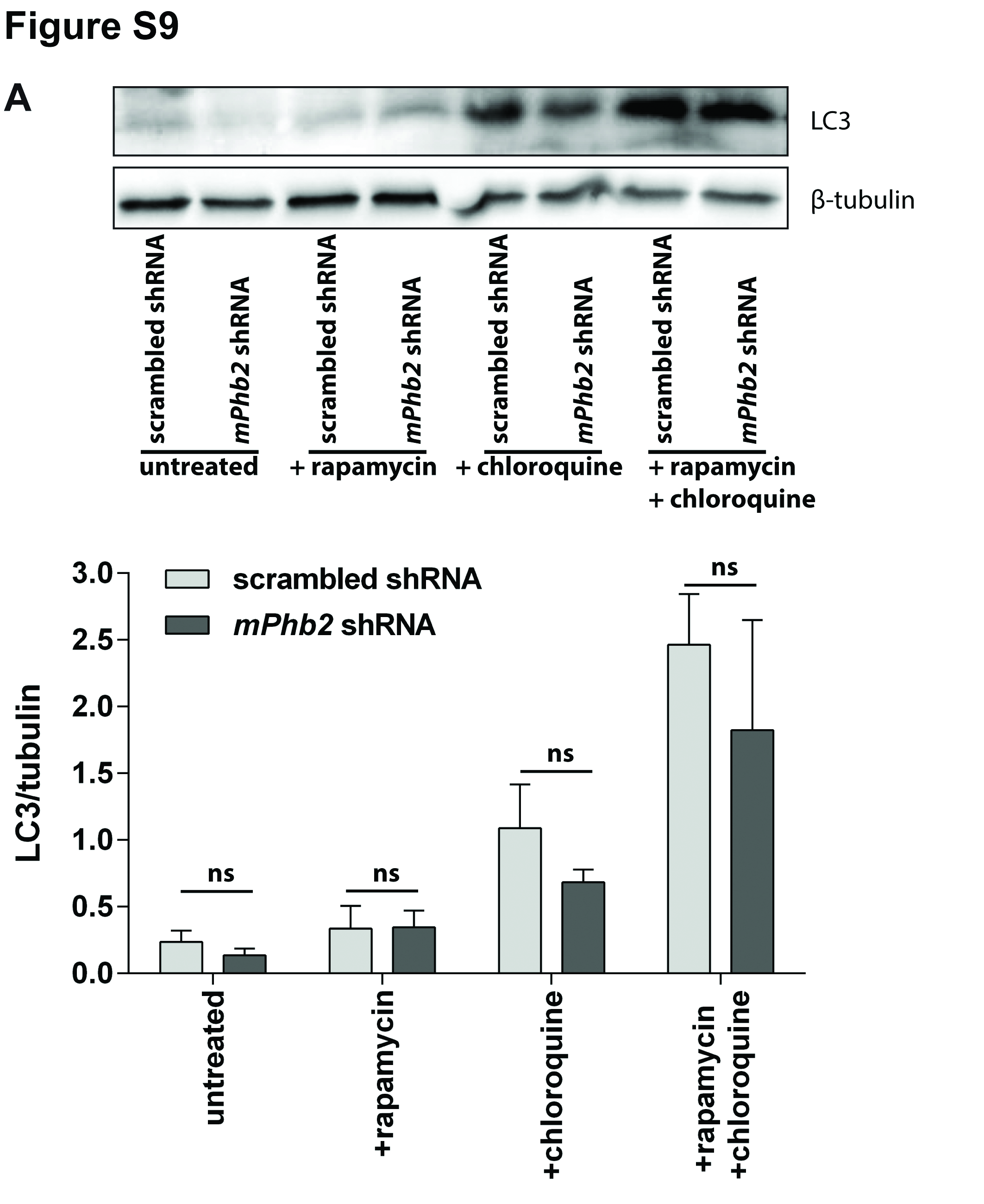

Supplement: Supplementary file 9 [file emmm0007-0275-sd9.tif]
