# Supplementary material for: Inhibition of insulin/IGF-1 receptor signaling protects from mitochondria-mediated kidney failure
Source: EMBO Mol Med. 2015 Feb 2;7(3):275–87. doi: 10.15252/emmm.201404916 (PMC4364945; doi:10.15252/emmm.201404916)
Supplement: Supplementary file 10 [file emmm0007-0275-sd10.doc]

**Supplementary Figure Legends**

**Figure S1 - *Phb2^pko^* mice are born following Mendelian rules**

A Phb2 flox/flox;NPHS2.cre mice are born following Mendelian rules (*Phb2^fl/wt^*, Phb2 flox/wt;NPHS2.cre wt/wt; *Phb2^fl/fl^*, Phb2 flox/flox;NPHS2.cre wt/wt; *Phb2^het^*, Phb2 flox/wt;NPHS2.cre tg/wt; *Phb2^pko^*, Phb2 flox/flox;NPHS2.cre tg/wt).

**Figure S2 - Glomeruli of *Phb2^pko^* mice display no increased rate of apoptosis at day 21.**

A Immunohistochemistry for cleaved caspase-3 on kidney sections of *Phb2^pko^* and *Phb2^fl/fl^* mice (scale bar 20 µm).

B Immunohistochemistry for the podocyte marker WT-1 on kidney sections of *Phb2^pko^* and *Phb2^fl/fl^* mice (scale bar 20 µm).

C Quantification of WT-1-positive cells per glomerulus (*n* = 3 for both groups, 40 glomeruli per animal counted, bars represent mean ± SEM, *P* = 0.4995).

**Figure S3 - Podocyte-specific double and triple-knockout mice develop albuminuria.**

A Representative coomassie stain of urinary samples of podocyte-specific double knockout‑mice.

B Representative coomassie stain of urinary samples of podocyte-specific triple-knockout mice (x = no urine analyzed because mouse was dead by that time).

**Figure S4 -** ***Insr^pko^/Igf1r^pko^* mice are healthy**

A Survival rate of *Insr^pko^/Igfr1^pko^* mice at day 55 was comparable to control mice (*n* = 4 for *Insr^pko^/Igfr1^pko^*, *n* = 4 for *Insr^fl/fl^/Igfr1^fl/fl^*, bars represent mean ± SEM).

B Coomassie stain of urinary samples of *Insr^pko^/Igfr1^pko^* mice revealed no albuminuria until week 5.

**Figure S5 – Validation of inducible mouse *Phb2*-knockdown podocytes**

A TaqMan® assay showed decreased mRNA expression of m*Phb2* but not m*Phb1* in *Phb2*-deficient (*mPhb2* shRNA) podocytes while expression levels of both were unchanged in control (scrambled shRNA) podocytes (*n* = 3, bars represent mean ± SEM, P = 0.0004).

B Western Blot analysis of PHB1 and PHB2 levels in *mPhb2* shRNA and control podocytes detected decreased levels of both proteins in *Phb2*-deficient podocytes. Staining for 14-3-3 used as loading control.

**Figure S6 – Loss of signaling via insulin and IGF-1 receptor decreases mTOR signaling in *Phb2*-deficient podocytes**

A Immunofluorescence for pS6RP on kidney sections of 28 days old *Phb2^fl/fl^, Phb2^pko^* and *Phb2^pko^/Insr^pko^/Igf1r^pko^* mice showed decreased phosphorylation of S6RP in glomeruli of *Phb2^pko^/Insr^pko^/Igf1r^pko^* mice (scale bar 20 µm).

B Western Blot analysis of *Phb2*-deficient mouse podocytes showed similar levels of phosphorylated S6 ribosomal protein (pS6RP) compared to control podocytes after treatment with a dual insulin receptor/IGF-1 receptor inhibitor (BMS 536924).

(pS6RP = phosphorylated S6 ribosomal protein, S6RP = S6 ribosomal protein)

**Figure S7 - Loss of *phb-2* leads to accelerated exit of DAF-16 from the nucleus after heat-shock**

A-D Bar plots depict the fraction of worms showing the specified subcellular localization of DAF-16::GFP at different timepoints after heat-shock (*n* = 4 for all points, bars represent mean ± SEM, *P* = 0.0085 for 15 minutes, *P* = 0.0001 for 30 and 45 minutes). Heat-shock results in the nuclear translocation of DAF-16. Depicted is the time course of cytoplasmic redistribution, a process known to be controlled by insulin receptor (DAF-2) signaling.

**Figure S8 - Vehicle-treated as well as rapamycin-treated *Phb2^pko^* mice develop albuminuria**

A Immunofluorescence for phosphorylated S6 ribosomal protein (pS6RP) on kidney sections of 21 days old rapamycin or vehicle-treated *Phb2^fl/fl^ and Phb2^pko^* mice revealed decreased phosphorylation of S6RP within glomeruli after treatment with rapamycin (scale bar 20 µm).

B Coomassie stain of urinary samples showed development of albuminuria in vehicle‑ as well as rapamycin-treated *Phb2^pko^* mice (x = no urine analysed because mouse was dead by that time).

**Figure S9 – *Phb2-*deficient podocytes show no significant changes in LC3 activation**

A *Phb2*-deficient mouse podocytes show no significant changes in LC3-accumulation (used as a marker for autophagosome formation) compared to control podocytes. Chloroquine was used as an inhibitor of autophagosome degradation to ease the quantification (*n* = 3, bars represent mean ± SEM, results not significant for all treatments when scrambled shRNA podocytes were compared to *Phb2*-deficient podocytes).

**Supplementary Materials and Methods**

**DAF-16::GFP nuclear localization**

*C. elegans* strains were maintained according to standard methods (Brenner, 1974) and grown on OP50 feeding bacteria. RNAi by feeding was performed as described by Fire et al. (Fir*e et* al, 1998). RNAi clones for *phb-2* and *daf-2* knockdown were used as contained in the library established by J. Ahringer’s laboratory (Kamath & Ahringer, 2003). The identity of the clones was confirmed using Sanger sequencing. After lysis of gravid hermaphrodites (TJ356 (Henderson & Johnson, 2001)) the eggs were placed on knockdown plates seeded with HT115 feeding bacteria harboring the respective RNAi plasmids. Upon reaching early adulthood 10-20 worms were placed on new knockdown dishes and transferred to 35°C for 2 hours. After returning the plates to 20°C subcellular localization of the transgene was monitored at the specified time points using an AxioZoom.V16 microscope (Carl Zeiss MicroImaging GmbH, Jena, Germany).

**qPCR for knockout efficiency**

Glomeruli from mice were isolated as described previously (Brähle*r et* al, 2012) and processed to single cell suspensions as described by Boerries et al. (Boerrie*s et* al, 2013).

Cells from glomerular cell suspensions were lysed using the QuantiLyse reagent (Pierc*e et* al, 2002). An equivalent of 100 cells was used per 20ul qPCR reaction. qPCR was performed using TaqMan Gene Expression Master (LifeTechnologies, Carlsbad, CA) on the ABI 7900HT System and analyzed using the ExpressionSuite software package (LifeTechnologies, Carlsbad, CA) applying the 2^-ΔΔCt^ method. Significance was calculated using a two-tailed student’s t-test in the Prism5 software (GraphPad). Probe-based qPCR assays were designed to detect exclusively either the WT *Phb2* allele or the respective allele after cre-mediated excision. An assay detecting *Tfrc* was used as an endogenous control. The following assays were ordered from IDTDNA and mixed at a primer:probe ratio of 2:1 :

m*Tfrc*

probe 5'-/5HEX/ACC ACA CCC /ZEN/AGC AAA GTA GGC TC/3IABkFQ/-3'

primer1 CCTTGAACTCAGATCCACCTG

primer2 TCAAACTCAGTGATCCACCTG

m*Phb2* WT

probe 5'-/56-FAM/CCC ATG TAC /ZEN/ACA CCG AGG CAA GT/3IABkFQ/-3'

primer1 5'-CCA CAA ACA GCA ACA GAG AAG-3'

primer2 5'-TTT GAG ATT CCC CAA GCC TG-3'

m*Phb2* MUT

probe 5'-/56-FAM/AGG ACA GTG /ZEN/GCT GCC TGA AAC TC/3IABkFQ/-3'

primer1 5'-CTT AAA CCC CTG ATC CCC TTG-3'

primer2 5'-CTG CTT TAC CCC TTC CTC AC-3'

**Mitochondrial respiration assay**

Mitochondrial oxygen consumption was measured as previously described (Mourie*r et* al, 2014), but at 33°C using 4-6 million cells re-suspended in mitochondrial respiratory buffer (120mM sucrose, 50mM KCl, 20mM Tris-HCl, 4mM KH2PO4, 2mM MgCl2, 1mM EGTA, pH7.2) in an oxygraph chamber (OROBOROS). Oxygen consumption was measured after digitonin permeabilization of the cells in presence of 10mM succinate and 10mM glycerol-3-phosphate as substrates of complex II and glycerol-3-phosphate dehydrogenase of the respiratory chain, and 1mM ADP (state 3) or 1mM ADP and 2.5μg/ml oligomycin (pseudo state 4). Respiration was then uncoupled by successive addition of carbonyl cyanide m-chlorophenyl hydrazone (CCCP) up to 3μM to reach maximal respiration (state 4 CCCP). Oxygen flux was normalized to cell amount.

**Mitochondrial DNA Quantification**

Total DNA was extracted using the DNeasy Blood & Tissue Kit (Qiagen). Relative mtDNA copy numbers were determined by analysis of the difference in cycle threshold between mtDNA and nuclear DNA (delta delta CT method) as described previously (Bari*s et* al, 2011). Primer pair mtDNA: sense CCTATCACCCTTGCCATCAT, antisense GAGGCTGTTGCTTGTGTGAC; primer pair for amplification of the Pecam gene, nuclear DNA: sense ATG GAAAGCCTGCCATCATG, antisense TCCTTGTTGTTCAGCATCAC.

**FACS analysis of mouse podocytes**

FACS analysis was performed with undifferentiated podocytes at 33°C. 10^6^ cells were detached with Collagenase II for 1 hour at 37°C and washed once with PBS and once with cell culture medium. After incubating the cells with either 50 nM MitoTracker Deep Red or 5 µM MitoSOX Red (both from Invitrogen, Carlsbad, CA) at 37°C for 15-30 minutes they were washed in FACS-buffer once and the pellet resuspended in 200 µl FACS-buffer. Analysis was done with a FACS Aria III with DIVA 6.0 software.

**References**

Boerries M, Grahammer F, Eiselein S, Buck M, Meyer C, Goedel M, Bechtel W, Zschiedrich S, Pfeifer D, Laloë D et al (2013) Molecular fingerprinting of the podocyte reveals novel gene and protein regulatory networks. *Kidney Int* 83: 1052 – 1064

Brähler S, Ising C, Hagmann H, Rasmus M, Hoehne M, Kurschat C, Kisner T, Goebel H, Shankland S, Addicks K et al (2012) Intrinsic proinflammatory signaling in podocytes contributes to podocyte damage and prolonged9 proteinuria. *Am J Physiol Renal Physiol* 303: F1473 – F1485

Brenner S (1974) The genetics of Caenorhabditis elegans. *Genetics* 77: 71 – 94

Fire A, Xu S, Montgomery MK, Kostas SA, Driver SE, Mello CC (1998) Potent and specific genetic interference by double-stranded RNA in Caenorhabditis elegans. *Nature* 391: 806 – 811

Henderson ST, Johnson TE (2001) daf-16 integrates developmental and environmental inputs to mediate aging in the nematode Caenorhabditis elegans. *Curr Biol* 11: 1975 – 1980

Kamath RS, Ahringer J (2003) Genome-wide RNAi screening in Caenorhabditis elegans. *Methods* 30: 313 – 321

Mourier A, Ruzzenente B, Brandt T, Kühlbrandt W, Larsson N-G (2014) Loss of LRPPRC causes ATP synthase deficiency. *Hum Mol Genet* 23: 2580 – 2592

Pierce KE, Rice JE, Sanchez JA, Wangh LJ (2002) QuantiLyse: reliable DNA amplification from single cells. *Biotechniques* 32: 1106 – 1111
