# Supplementary material for: Inhibition of insulin/IGF-1 receptor signaling protects from mitochondria-mediated kidney failure
Source: EMBO Mol Med. 2015 Feb 2;7(3):275–87. doi: 10.15252/emmm.201404916 (PMC4364945; doi:10.15252/emmm.201404916)
Supplement: Supplementary file 11 [file emmm0007-0275-sd11.pdf]

Inhibition of insulin/IGF-1 receptor signaling protects from mitochondria-mediated kidney failure

Christina Ising, Sybille Koehler, Sebastian Brähler, Carsten Merkwirth, Martin Höhne, Olivier R. Baris, Henning Hagmann, Martin Kann, Francesca Fabretti, Claudia Dafinger, Wilhelm Bloch, Bernhard Schermer, Andreas Linkermann, Jens C. Br¸ning, Christine E. Kurschat, Roman-Ulrich M¸ller, Rudolf J. Wiesner, Thomas Langer, Thomas Benzing, Paul Thomas Brinkkoetter

Corresponding author: Thomas Benzing, Uniklinik Köln

Review timeline:

Submission date to The EMBO Journal:	07 May 2014
Editorial Decision:	03 June 2014
Revision received:	14 November 2014
Editorial Decision:	02 December 2014
Transfer to EMBO Molecular Medicine	02 December 2014
Editorial Decision:	02 December 2014
Revision received:	16 December 2014
Editorial Decision:	18 December 2014
Revision received:	30 December 2014
Accepted:	08 January 2015

Transaction Report:

(Note: With the exception of the correction of typographical or spelling errors that could be a source of ambiguity, letters and reports are not edited. The original formatting of letters and referee reports may not be reflected in this compilation.)

Editor: Céline Carret

1st Editorial Decision 03 June 2014

Thank you very much for submitting your study that reveals a disease-relevant crosstalk between Insulin/IGF-1/mTOR-signaling and prohibitin's structural function in kidney podocytes (and possibly renal failure) for consideration at The EMBO Journal.

I enclose the comments from three scientists that appreciate novel findings with certain translational relevance. Their detailed assessments commend you on one hand to these genetically rather comprehensive results. On the other, and reflecting from the perspective and scope of The EMBO Journal, they demand further mechanistic detail that should go beyond relevant results recently published on mTOR-activity in alleviating mitochondrial diseases.

While we are very much interested to further pursue your study, we realize that significant and demanding further experimental work as specified in ref#2 points 1 and 3 should be performed to eventually meet the expectations at our relatively molecular-oriented title. The specific experiments proposed from this expert scientist appear very suited to generate such advance.

Further, I encourage you to provide necessary controls for the efficiency of Cre-mediated deletion, preferably in the in-vivo situation (and compared to ref#1's suggestion to use the complementary

cellular system). The latter could however become very useful to study and gauge novel mechanistic insights.

Lastly, we do agree with the already supportive ref#3 that the worm data are not adding much to the major story-line, thus could either be left out in a thoroughly revised paper OR presented as further supporting evidence in an expanded-view figure.

Clearly conditioned on the outlined crucial amendments, we are prepared to offer the opportunity to develop the current dataset for a subsequent peer-review assessment at The EMBO Journal. This will have to involve refs#1 and#2 for a balanced response.

Please do not hesitate to get in touch (due to time constrains and the complexity of the matter preferably via e-mail) in case further questions arise OR to discuss anticipated timeline/feasibility of certain experiments.

I am sorry to be unable to communicate more enthusiastic news at least at this point and remain with best regards.

REFEREE REPORTS:

Referee #1:

This paper presents evidence that knocking down the mitochondrial membrane protein, prohibitin ring complex subunit-2 (PHB2) specifically in the podocyte of the kidney causes breakdown of the glomerular filtration barrier, albuminuria, glomerulosclerosis, renal failure and death in a podocyte specific transgenic mouse model. They go on to show that this occurs also in an inducible transgenic model suggesting this is not only a developmental phenomenon. The authors then suggest that knocking down the insulin and IGF-1 signaling pathway to this cell partially rescues the phenotype by crossing in 4 further transgenes (floxed insulin- homozygous and floxed IGF-1 Receptor-homozygous). By doing this they find a survival benefit (but no benefit for albuminuria) for the insulin receptor and the double insulin receptor, IGF-I receptor mice but not for the IGF-I receptor mice alone. Finally they examine the role of mTOR signaling in this pathway and show hyperphosphorylation of S6RP (a down stream effector of mTOR) in the PHB2 knockout/down mice. They are able to do this by generating a PHB2 knock down cell line of podocytes (which also knocked down PHB1) and showed it is up-regulation. They then gave the PHB2 podocyte KO mice rapamycin and rescued improved survival but, again, did not alter albuminuria. They also performed a heat shock experiment in C. Elegans in which PHB2 was knocked down and this showed improved DAF-16 cytoplasmic localisation. This implies that knocking down this mitochondrial protein results in hyperactive insulin/igf signaling.

I think this is interesting work with some novel findings. However, there are a number of major weaknesses that need to be addressed.

Major issues

1. Loss of PHB2 in the podocyte is detrimental.
I think to a general readership this is not surprising as it has been shown that knocking this molecule out in the whole body is embryonic lethal and when knocked out in the neurones it causes a progressive neuropathy, neuronal cell death and mortality by 24 weeks (Merkwirth 2012 as stated in paper). Therefore this aspect of the data, I think, will be interesting predominantly for kidney-orientated researchers.

2. Although there is evidence of genetic loss of PHB2, the insulin receptor (IR) and the IGF1Receptor (IGF1R) using genomic PCR analysis of tail cuts, in the transgenic mouse model, there is no evidence that these genes and proteins have been depleted in the podocyte in this model. I think this is critical information for the podocyte specific PHB2/ Insulin receptor / IGF1R models and combined models. One obvious possibility is that by adding in extra transgenes into this model that the level of cre mediated PHB2 knockdown in the podocyte is simply being reduced- and this is

resulting in improved survival. It is essential to show that in vivo in this model that the genes/proteins are adequately knocked down in the podocyte and the insulin/ IGF signaling pathways are being abrogated in this cell. Following on from this it is also plausible that this is a model of partial podocyte insulin /IGF resistance in a model of mitochondria dysfunction, and that this is having a beneficial effect. It is extensively described that partial resistance to IGF and IR signaling is beneficial for long term survival with respect to oxidative stress and mitochondrial function (e.g. Holzenberger et al Nature 421, 182-187 (2003) / Zarse et al Cell Metab. 2012 Apr 4;15(4):451-65). However in this study it is impossible to decipher this potential mechanism due to a lack of data. It is interesting / surprising that knocking down the IGF1R alone (which will take out the IGF signaling pathway) does not effect survival. As is the fact that it is proposed that complete combined loss of Insulin and IGF signalling to this cell is compatible with improved cell survival.

3. Further to point 2 an in vivo experiment may help, if it has not been performed yet. The conditionally immortalized podocytes described in the paper could knock down PHB2 (as performed), but also the insulin receptor and the IGF1 receptor, and all 3 genes. The level of knock down could be ascertained and the functional significance of knock down assessed. This may reveal the survival benefits of inhibiting the PHB2, insulin and IGF pathways in this model. This would support the underlying hypothesis of this work. Currently it appears that only PHB2 has been examined in this fashion.

4. There is very little mechanism presented as to how the presumed mitochondrial dysfunction causes altered insulin and mTOR signalling. This would be helpful for an excellent general journal like this.

5. There is no mechanism or explanation why there is no difference in the profound albuminuria when the extra genes are introduced into the system. Are these through different mechanisms? Also it is not clear if there is a difference in renal function when the insulin and or IGF1 receptor are also knocked down. This would be useful.

6. This group has recently published an abstract in the JASN ([SA-OR028] Loss of Prohibitin-2 in Podocytes Leads to Proteinuria and Renal Failure in Mice) which states that PHB2 is also expressed in the slit diaphragm which was presented at this years American Society of Nephrology. In it they described that PHB2 is also expressed in the slit diaphragm of the podocyte, as well as mitochondria, and it is a structural protein here associated with podocin. This is an extension of previous work from this laboratory (Huber et al Proc Natl Acad Sci U S A. Nov 14, 2006; 103(46): 17079-17086). I wonder why this data is not included here and if it is relevant to the albuminuric phenotype? I think this may be important for mechanism.

Referee #2:

This is an interesting manuscript that reports the beneficial role of IGF and mTOR inhibition on glomerular dysfunction caused by knocking out the mitochondrial protein prohibitin (Phb). Using refined in vivo models, the authors show that podocyte specific Phb deletion is lethal and causes kidney failure, which is partially rescued by ablation of the Igf signaling and by mTOR inhibition. The authors conclude that a component of kidney failure caused by mitochondrial dysfunction can be treated by interfering with Igf and mTOR.

While the manuscript reports some interesting finding on the role of mitochondria in kidney failure and of the interplay with other signaling cascades, at this stage the mechanistic insight is too limited to warrant publication in EMBOJ and the paper is more suited for a clinically oriented journal. However, the authors could perform several experiments that would address the key underlying mechanistic questions and add the required insight for this journal. More specifically,

1. the mechanism of glomerular dysfunction caused by Phb deletion is not addressed. Are mitochondria dysfunctional? does this lead to cell death? is this cell death necrotic or apoptotic? is

autophagy activated (see also below). These fundamental questions shall be addressed in vivo as well as in an in vitro model of Phb silencing in a podocyte cell line

2. these authors have extensively published on the Phb knockout in other tissues. Are the effects of Phb deletion in podocytes the same as the ones observed in the neurons, for example?

3. the reader is left with the unanswered question of what is the mechanism by which rapamycin protects the Phb knockouts. Since this drug is a well characterized inducer of autophagy, it could be that the dysfunctional mitochondria are eliminated, or that other signals emanating from mitochondria are similarly deleted. Experiments to address (i) autophagy and autophagic flux; (ii) mitochondrial morphology and number; (iii) mitochondrial ultrastructure in the treated single and in the triple knockouts are missing and deem required by this reviewer to understand the underlying mechanism explaining the observed phenotype of protection. The experiments with phosphorylated S6 just tell us that mTOR is activated in Phb deficient podocytes and that as expected knocking out Igf restores this, but do not enlighten the reader on the mechanism by which this happens.

4. authors state that their data complement the Johnson et al 2013 ones. However, to reach the level for EMBOJ they shall add something new, like for example they shall dissect whether rapamycin acts via or independently from mitophagy.

In conclusion, while the paper represents a commendable technical in vivo tour de force, it falls short addressing the key question of why rapamycin (and IGF signaling inhibition) is beneficial for podocytes lacking Phb.

Referee #3:

In this manuscript Ising et al report that podocyte specific knockout of the Phb2 results in severe kidney disease that is partially suppressed by deletion of the insulin and IGF-1 receptors or by pharmacological treatment with the mTOR inhibitor rapamycin. This is an important study because it demonstrates a causal link between mitochondrial dysfunction and kidney disease as well as a potential therapeutic approach in the form of rapamycin or other mTOR inhibitors. It also further supports a direct role for mTOR hyperactivation in mitochondrial disease. I have only a few relatively minor suggestions.

Specific comments:

1. I'm not sure why the authors emphasize insulin/IGF-1 signaling in the title and abstract, when they have identified a specific molecular target (mTOR) that appears to account for the effects in this model. Although mTOR activity is certainly influenced by insulin/IGF-1-like signaling (and vice versa), there are insulin/IGF-1-independent ways to regulate mTOR (rapamycin for one), and I think it is an oversimplification to consider mTOR as part of the "insulin/IGF-1 pathway". I would suggest changing the title/abstract to reflect the importance of mTOR specifically and revising the text in the relevant places.

2. I found the data in Fig E5 on DAF-16 relocalization to be somewhat distracting and not particularly relevant. It's not clear what this is actually reflecting or that the mechanisms are at all related to mTOR hyperactivation in the phb2 deficient mice. The worm data is not needed to further support the mouse data, as the P-S6 is clear indication for mTOR hyperactivation.

3. Similar hyperactivation of mTOR in mitochondrial dysfunction mouse and zebrafish models has been previously reported by Johnson et al. [1] and Kim et al. [2]. This should be mentioned somewhere in the text.

4. Some of the survival curves in Fig 4 are hard to see due to the colors used. The mortality kinetics for the phb2 insr igf1r animals are interesting, as it appears that about half of the animals obtained

little benefit and half a very large benefit. Any idea what this means?

5. Johnson et al. observed much greater effects on survival in the Ndufs4 KO mice at 8 mg/kg/day rapamycin compared to half that dose [1]. I wonder if much larger effects would also be seen in this model at a similar dose of rapamycin. It's not necessary to do this experiment, as the effect is clear even at the lower dose used here, but perhaps should be noted in the discussion.

References:

[1] S.C. Johnson, M.E. Yanos, E.B. Kayser, A. Quintana, M. Sangesland, A. Castanza, L. Uhde, J. Hui, V.Z. Wall, A. Gagnidze, K. Oh, B.M. Wasko, F.J. Ramos, R.D. Palmiter, P.S. Rabinovitch, P.G. Morgan, M.M. Sedensky, M. Kaeberlein, mTOR inhibition alleviates mitochondrial disease in a mouse model of Leigh syndrome, Science 342 (2013) 1524-1528.
[2] S.H. Kim, S.A. Scott, M.J. Bennett, R.P. Carson, J. Fessel, H.A. Brown, K.C. Ess, Multi-organ abnormalities and mTORC1 activation in zebrafish model of multiple acyl-CoA dehydrogenase deficiency, PLoS Genet 9 (2013) e1003563.

1st Revision - authors' response 14 November 2014

Referee #1:

1) Loss of PHB2 in the podocyte is detrimental. I think to a general readership this is not surprising as it has been shown that knocking this molecule out in the whole body is embryonic lethal and when knocked out in the neurones it causes a progressive neuropathy, neuronal cell death and mortality by 24 weeks (Merkwirth 2012 as stated in paper). Therefore, this aspect of the data, I think, will be interesting predominantly for kidney-orientated researchers.

We appreciate the reviewer's careful evaluation of our manuscript. We feel that this work is interesting to a broad readership for a number of reasons. Firstly, as referenced by the referee and also in the manuscript the conventional knockout of PHB2 is lethal (Merkwirth et al., 2008). However, the underlying mechanism still remained elusive. Here, we now link PHB2-dependent mitochondrial dysfunction with intracellular signaling cascades and deregulated insulin/IGF1 and mTOR activity. Therefore, this work connects mitochondrial fusion/fission defects with recent exciting new data on classic mitochondrial respiratory dysfunction and deregulated mTOR signaling. Kaeberlein and colleagues (Johnson et al, 2013) recently demonstrated that respiratory chain defects can lead to the activation of mTOR signaling. Moreover, Kim et al. (Kim et al, 2013) showed that defects in mitochondrial β-oxidation activated the mTOR pathway. Taken together, all these studies point to a previously underappreciated role of hyperactive mTOR signaling in mitochondrial disease. Secondly, this manuscript contains an intervention study. Almost all work that has been published on podocyte defects and kidney disease or genetically-induced mitochondrial dysfunction in general described mechanisms and phenotypes but did not suggest potential therapeutic interventions. Here, we show that both genetic (generation of triple knock-out animals) as well as pharmacologic intervention did not only revert the cellular phenotypes but was suitable to ameliorate (and treat) disease and prevent death from endstage renal disease. Thus, this study may suggest new treatment options for a variety of mitochondria-mediated disorders and may therefore provide a starting point for clinical translation. We feel that these issues are very important for a general readership. To better make our point we have modified the discussion in our revised manuscript.

2) Although there is evidence of genetic loss of PHB2, the insulin receptor (IR) and the IGF1Receptor (IGF1R) using genomic PCR analysis of tail cuts, in the transgenic mouse model, there is no evidence that these genes and proteins have been depleted in the podocyte in this model. I think this is critical information for the podocyte specific PHB2/ Insulin receptor / IGF1R models and combined models. One obvious possibility is that by adding in extra transgenes into this model that the level of cre mediated PHB2 knockdown in the podocyte is simply being reduced- and this is resulting in improved survival. It is essential to show that in vivo in this model that the

genes/proteins are adequately knocked down in the podocyte and the insulin/ IGF signaling pathways are being abrogated in this cell. Following on from this it is also plausible that this is a model of partial podocyte insulin /IGF resistance in a model of mitochondria dysfunction, and that this is having a beneficial effect. It is extensively described that partial resistance to IGF and IR signaling is beneficial for long term survival with respect to oxidative stress and mitochondrial function (e.g. Holzenberger et al Nature 421, 182-187 (2003) / Zarse et al Cell Metab. 2012 Apr 4;15(4):451-65). However in this study it is impossible to decipher this potential mechanism due to a lack of data. It is interesting / surprising that knocking down the IGF1R alone (which will take out the IGF signaling pathway) does not effect survival. As is the fact that it is proposed that complete combined loss of Insulin and IGF signaling to this cell is compatible with improved cell survival.

We thank the reviewer for the valuable comments and agree that this is a very important point. Although we had already initially carefully tested for *Cre* efficiency in the various combined knock-out models, we have now performed additional experiments that assure efficient *Cre*-mediated gene deletion in the double and triple floxed animals. We studied the mitochondrial ultrastructure in podocytes from $Phb2^{pko}/Insr^{pko}/Igf1r^{pko}$ mice as compared to $Phb2^{pko}/Insr^{wt}/Igf1r^{wt}$ animals by EM. $Phb2^{pko}$ animals display severely altered mitochondria with disrupted mitochondrial ultrastructure. Although combined deletion of PHB2 and IR/IGF1R ($Phb2^{pko}/Insr^{wt}/Igf1r^{wt}$) prevented mTOR hyperactivity it did not prevent these severe mitochondrial alterations. In fact, $Phb2^{pko}/Insr^{wt}/Igf1r^{wt}$ and $Phb2^{pko}/Insr^{pko}/Igf1r^{pko}$ podocytes were indistinguishable with regard to mitochondrial morphology (new Fig. 5C) suggesting that combined gene deletion of *Phb2* and *Insr/Igf1r* did not affect *Phb2* gene deletion efficiency.

In addition, we purified genomic DNA from isolated glomeruli from 2 weeks old $Phb2^{pko}$ and $Phb2^{pko}/Insr^{pko}/Igf1r^{pko}$ mice and performed quantitative PCR specifically detecting the deleted *Phb2* allele. Quantitative assessment of *Cre* recombinase efficiency in $Phb2^{pko}$ as compared to $Phb2^{pko}/Insr^{pko}/Igf1r^{pko}$ mice revealed comparable gene deletion (new Fig. 5B) clearly indicating that differences in gene deletion efficiency cannot account for the altered mTOR activity or milder phenotype in triple knock-out animals.

3) Further to point 2 an in vitro experiment may help, if it has not been performed yet. The conditionally immortalized podocytes described in the paper could knock down PHB2 (as performed), but also the insulin receptor and the IGF1 receptor, and all 3 genes. The level of knock down could be ascertained and the functional significance of knock down assessed. This may reveal the survival benefits of inhibiting the PHB2, insulin and IGF pathways in this model. This would support the underlying hypothesis of this work. Currently it appears that only PHB2 has been examined in this fashion.

We appreciate the reviewers comment. However, we were unable to efficiently knock down all three genes in our conditionally immortalized podocyte cell line due to technical limitations. These cells are very hard to transfect impeding combined knock-down of several genes. Since we also thought that the reviewer's idea would strengthen the conclusions of our study we used an alternative strategy and treated the *Phb2*-deficient podocytes with BMS 536924 (from Tocris), an inhibitor specific for the insulin and IGF1 receptors. This compound has been widely used to study insulin/IGF signaling in mammalian tissues and the concentrations of BMS that we chose was comparable to the published data (Wahner, Hendrickson et al, 2009; Wittman et al, 2005). Very similarly to the *in vivo* data, treatment of *Phb2*-deficient podocytes with this inhibitor prevented hyperphosphorylation of S6 ribosomal protein induced by PHB2 deficiency supporting a direct link between mitochondrial dysfunction, hyperactive IR/IGF1R signaling and downstream mTOR hyperactivity. This data is now included in new Fig. E6B.

4) There is very little mechanism presented as to how the presumed mitochondrial dysfunction causes altered insulin and mTOR signaling. This would be helpful for an excellent general journal like this.

We took this advice very seriously and performed a series of additional experiments that are depicted in an entire set of new figures in the revised paper. To expand our mechanistic data on how mitochondrial dysfunction may cause altered insulin/mTOR signaling we generated a cell culture system which allowed inducible Phb2 knock-down in podocytes in culture. We used this cell culture model to analyse mitochondrial function and to measure oxygen consumption, ROS production as well as mitochondrial mass. As expected, mitochondrial structure was severely altered in *Phb2* knockdown cells which we analysed further using TOM-20 stainings (Fig. 6A). Mitochondria were fragmented (Fig. 6B) and mitochondrial size and branching were significantly decreased while the number of mitochondria per area was increased in Phb2-deficient podocytes (new Fig. 6C-E). However, we did not observe any significant effects of *Phb2* gene deletion on respiratory chain activity (oxygen consumption) or ROS production or mitochondrial DNA content (new Fig. 6F-I). These results indicated that rather a cellular signaling program than a mitochondrial respiratory function defect may account for the severe podocyte dysfunction and cell loss. How this mitochondrial morphological defect may cause altered insulin and IGF-1 receptor signaling and hyperactive mTOR remained elusive. However, the defects could not be attributed to classic mitochondrial respiratory function but rather appear the result of a detrimental metabolic switch induced by the mitochondrial structural defect.

5) There is no mechanism or explanation why there is no difference in the profound albuminuria when the extra genes are introduced into the system. Are these through different mechanisms? Also it is not clear if there is a difference in renal function when the insulin and or IGF1 receptor are also knocked down. This would be useful.

This group has recently published an abstract in the JASN ([SA-OR028] Loss of Prohibitin-2 in Podocytes Leads to Proteinuria and Renal Failure in Mice) which states that PHB2 is also expressed in the slit diaphragm which was presented at this years American Society of Nephrology. In it they described that PHB2 is also expressed in the slit diaphragm of the podocyte, as well as mitochondria, and it is a structural protein here associated with podocin. This is an extension of previous work from this laboratory (Huber et al Proc Natl Acad Sci U S A. Nov 14, 2006; 103(46): 17079-17086). I wonder why this data is not included here and if it is relevant to the albuminuric phenotype? I think this may be important for mechanism.

As stated in the initial version of the paper, loss of insulin/IGF-1 receptor signaling alleviated renal disease in Phb^{pko} mice and delayed the onset of renal failure resulting in improved survival (Fig. 4A-E). We observed a significantly improved renal function in Phb2-deficient mice harbouring an *Insr/Igf1r* gene defect as compared to *Phb2*-deficient and *Insr/Igf1r*-proficient mice (Fig. 5A-B). Based on these findings we hypothesize that two different mechanisms may contribute to the phenotype that are being separated experimentally. First, hyperactivation of the insulin/mTOR pathway as a result of mitochondrial dysfunction may cause podocyte loss, glomerulosclerosis and renal failure. This abnormality is amenable to genetic intervention in the *Insr/Igf1r*-deficient animals or to therapeutic intervention with mTOR inhibitor. However, the severity of albuminuria may also be affected by mitochondria-independent functions of PHB2 (that cannot be alleviated by mTOR inhibition). In fact, preliminary data suggests that PHB2 is not only localized to the mitochondrial inner membrane but may also be present at the slit diaphragm protein complex at the filtration barrier of the kidney. However, much more work is needed to validate these findings and address additional, mitochondria-independent roles of the protein which cannot be separated in *in vivo* experiments. These studies are ongoing but far beyond the scope of this manuscript.

References for Referee #1:

Johnson SC, Yanos ME, Kayser E-B, Quintana A, Sangesland M, Castanza A, Uhde L, Hui J, Wall VZ, Gagnidze A, Oh K, Wasko BM, Ramos FJ, Palmiter RD, Rabinovitch PS, Morgan PG, Sedensky MM & Kaeberlein M (2013) mTOR inhibition alleviates mitochondrial disease in a mouse model of Leigh syndrome. *Science* **342:** 1524–1528

Kim S-H, Scott SA, Bennett MJ, Carson RP, Fessel J, Brown HA & Ess KC (2013) Multi-organ abnormalities and mTORC1 activation in zebrafish model of multiple acyl-CoA dehydrogenase deficiency. *PLoS Genet.* **9:** e1003563

Wahner Hendrickson AE, Haluska P, Schneider PA, Loegering DA, Peterson KL, Attar R, Smith BD, Erlichman C, Gottardis M, Karp JE, Carboni JM & Kaufmann SH (2009) Expression of insulin receptor isoform A and insulin-like growth factor-1 receptor in human acute myelogenous leukemia: effect of the dual-receptor inhibitor BMS-536924 in vitro. *Cancer Res.* **69:** 7635–7643

Wittman M, Carboni J, Attar R, Balasubramanian B, Balimane P, Brassil P, Beaulieu F, Chang C, Clarke W, Dell J, Eummer J, Frennesson D, Gottardis M, Greer A, Hansel S, Hurlburt W, Jacobson B, Krishnananthan S, Lee FY, Li A, et al (2005) Discovery of a (1H-benzoimidazol-2-yl)-1H-pyridin-2-one (BMS-536924) inhibitor of insulin-like growth factor I receptor kinase with in vivo antitumor activity. *J. Med. Chem.* **48:** 5639–5643

Referee #2:

1) The mechanism of glomerular dysfunction caused by Phb deletion is not addressed. (i) Are mitochondria dysfunctional? (ii) does this lead to cell death? is this cell death necrotic or apoptotic? (iii) is autophagy activated (see also below). These fundamental questions shall be addressed in vivo as well as in an in vitro model of Phb silencing in a podocyte cell line.

We thank the reviewer for the overall positive evaluation of our manuscript. We have performed all the experiments suggested by this reviewer which are now presented as new data in the revised manuscript.

(i) Are mitochondria dysfunctional?

To expand our mechanistic data on how mitochondrial dysfunction may cause altered insulin/mTOR signaling we generated a cell culture system which allowed inducible *Phb2* knock-down in podocytes in culture. We used this cell culture model to analyse mitochondrial function and to measure oxygen consumption, ROS production as well as mitochondrial mass. These data are now added in the original figures as well as in a number of supplemental figures in the paper. As expected, mitochondrial structure was severely altered in *Phb2* knockdown cells which we analysed further using TOM-20 stainings (Fig. 6A). Mitochondria were fragmented (Fig. 6B) and mitochondrial size and branching were significantly decreased while the number of mitochondria per area was increased in Phb2-deficient podocytes (new Fig. 6C-E). However, we did not observe any significant effects of *Phb2* gene deletion on respiratory chain activity (oxygen consumption) or ROS production (new Fig. 6F-I). These data indicated that loss of *Phb2* resulted in dysmorphic mitochondria without primarily affecting OXPHOS activity. How this mitochondrial morphological defect may cause altered insulin and IGF-1 receptor signaling and hyperactive mTOR remained elusive. However, our data clearly indicated that rather a cellular signaling program than a mitochondrial respiratory function defect may account for the severe podocyte dysfunction and cell loss.

(ii) does this lead to cell death? is this cell death necrotic or apoptotic?

We appreciate the reviewer's important questions. Podocytes have a limited ability of self renewal. As discussed in our initial version of the manuscript loss of podocytes clearly contributes to the phenotype observed in *Phb2*^{pko} mice (pathogenesis of glomerulosclerosis reviewed in (Shankland, 2006)). In principle, loss of podocytes can either result from cell death (apoptosis or necrosis) or detachment from the basement membrane and loss in urine (which has been demonstrated for a large variety of human diseases). To address the important question if podocytes may undergo apoptosis in our model we quantified the number of podocytes per glomerulus and the number of cells positive for an apoptotic marker at 3 weeks of age when the animals showed first signs of glomerular disease. Kidney sections of *Phb2*^{pko} and control mice were double stained for the podocyte marker WT-1 and the apoptotic marker cleaved caspase 3 (see Figure E2A-B). At this early time point no loss of podocytes was detectable indicating that the onset of disease does not involve podocyte cell death (podocyte number was not altered) or the development of apoptosis (no difference in cleaved

caspase 3 staining). However, the disease in this model progresses very rapidly. Already several days after the onset of proteinuria, the number of podocytes is dramatically decreased and glomerular scarring (glomerulosclerosis) develops. However, whether this was due to apoptotic or necrotic cell death or detachment from the underlying glomerular basement membrane could not be delineated. This issue is now carefully discussed in the revised manuscript.

(iii) is autophagy activated?

We have addressed this issue with a series of new experiments that are presented in our response to point 3 of Referee #2.

2. these authors have extensively published on the Phb knockout in other tissues. Are the effects of Phb deletion in podocytes the same as the ones observed in the neurons, for example?

This is an important question that we now discuss in more detail in the revised version of the manuscript. Briefly, we have previously shown that in neurons lacking *Phb2* gene expression mitochondrial structure is severely altered. Similarly to the situation in podocytes, mitochondria were fragmented and mitochondrial size and branching were significantly decreased. Moreover, *Phb2*-deficient neurons do not show defects in OXPHOS activity prior to 18 weeks after birth. However, we have not studied mTOR activity or altered insulin and IGF-1 receptor activity in these earlier studies. Furthermore, we have not performed the extensive genetic experiments (double and triple conditional gene deletions) that we present here.

3. the reader is left with the unanswered question of what is the mechanism by which rapamycin protects the Phb knockouts. Since this drug is a well characterized inducer of autophagy, it could be that the dysfunctional mitochondria are eliminated, or that other signals emanating from mitochondria are similarly deleted. Experiments to address (i) autophagy and autophagic flux; (ii) mitochondrial morphology and number; (iii) mitochondrial ultrastructure in the treated single and in the triple knockouts are missing and deem required by this reviewer to understand the underlying mechanism explaining the observed phenotype of protection. The experiments with phosphorylated S6 just tell us that mTOR is activated in Phb deficient podocytes and that as expected knocking out Igf restores this, but do not enlighten the reader on the mechanism by which this happens.

We thank Referee #2 for these highly relevant questions and experimental suggestions. We have performed all the required experiments to address these issues. These new experiments are now included in a revised version of the manuscript.

(i) experiments to address autophagy

Since active mTOR inhibits autophagy in podocytes (Inoki & Huber, 2012), we also quantified accumulation of the autophagosome marker LC3 in podocytes *in vitro.* Although a trend to less autophagy was seen in *Phb2*-deficient podocytes compared to control podocytes (new Figure E9A) this effect was not statistically significant. Even inhibition of autophagosomal degradation by adding chloroquine to the cells did not result in any significant differences in the accumulation of autophagosomes (new Figure E9A). This is in accordance with our electron microscopy studies where we did not observe a reduced amount of autophagosomes in *Phb2*^{pko} mice (not shown) suggesting that the mTOR effect may be independent of influencing the level of autophagy.

(ii) mitochondrial morphology and number

To better understand the beneficial effect of podocyte-specific insulin signaling deficiency for renal disease and the role of PHB2 on metabolic signaling in podocytes we generated a *Phb2* knockdown podocyte cell culture model. As *Phb2*-deficiency leads to a reduced cellular proliferation rate impeding the generation of conventional stable cell lines, we utilized a doxycycline-inducible promoter to express short hairpin RNAs directed against *Phb2* mRNA in mouse podocytes.

Knockdown of *Phb2* in podocytes resulted in a disrupted reticular mitochondrial network and the accumulation of fragmented mitochondria (Figure 6A-B). The mitochondrial morphology was further analyzed by using morphometric image analysis of the ratio of discrete mitochondria number to total mitochondrial area as a quantitative measure of mitochondrial length/interconnectivity. These data showed a significant difference between control and *Phb2* knockdown podocytes (new Figure 6C). Moreover, we detected less branches and smaller mitochondrial sizes after loss of PHB2 (new Figure 6D-E). However, these structural abnormalities were not accompanied by an impaired oxidative phosphorylation system. We did not detect any differences in oxygen consumption rates between *Phb2*-deficient and control podocytes using complex II substrates (succinate and glycerol-3-phosphate) (new Figure 6F). The same hold true when complex I substrates (pyruvate, glutamate and malate) were used (data not shown). Furthermore, there was no evidence for a compensatory upregulation of mitochondrial number as we did not detect differences in total mitochondrial mass by using both, quantification of mtDNA by qPCR (new Figure 6G) and FACS-analysis of podocytes stained with the mitochondrial marker MitoTracker (new Figure 6H-I). Additionally, we did not detect any change in reactive oxygen species (ROS) in *Phb2*-deficient podocytes (new Figure 6H-I). These findings are in accordance with our previous work showing an unaltered mitochondrial respiratory function after loss of PHB2 in MEFs (Merkwirth *et al*, 2008). These results indicated that rather a cellular signaling program than a mitochondrial respiratory function defect may account for the severe podocyte dysfunction and cell loss.

(iii) mitochondrial ultrastructure in the treated single and in the triple knockouts

To address the question if mitochondrial ultrastructure is different in our rescued animals we performed EM analysis on 3 weeks old $Phb2^{pko}/Insr^{pko}/Igf1r^{pko}$ mice. We observed the same ultrastructural abnormalities as seen in $Phb2^{pko}$ mice (new Figure 5C). These data clearly indicated that loss of insulin and IGF-1 receptor activity did not rescue the morphological defect although preventing the detrimental mTOR hyperactivity. Since mTOR activation is a downstream event in the insulin signaling cascade we think that an additional analysis of rapamycin-treated animals will not add much to this study. These data are now presented in the revised version of the manuscript.

4. authors state that their data complement the Johnson et al 2013 ones. However, to reach the level for EMBO J they shall add something new, like for example they shall dissect whether rapamycin acts via or independently from mitophagy.

This important thought is now addressed in the revised version of the manuscript. Neither our *in vitro* stainings for the autophagic marker LC3 nor the EM analysis of $Phb2^{pko}$ mice showed any significant effect of loss of *Phb2* on the rate of autophagy or mitophagy. Moreover, we added new complementing data by assessing mitochondrial mass as well as levels of reactive oxygen species (ROS). These data are described in our response to point 3 of Referee #2.

References for Referee #2:

Losón OC, Song Z, Chen H & Chan DC (2013) Fis1, Mff, MiD49, and MiD51 mediate Drp1 recruitment in mitochondrial fission. *Mol. Biol. Cell* **24:** 659–667

Shankland SJ (2006) The podocyte's response to injury: role in proteinuria and glomerulosclerosis. *Kidney Int.* **69:** 2131–2147

Referee #3:

1. I'm not sure why the authors emphasize insulin/IGF-1 signaling in the title and abstract, when they have identified a specific molecular target (mTOR) that appears to account for the effects in this model. Although mTOR activity is certainly influenced by insulin/IGF-1-like signaling (and vice versa), there are insulin/IGF-1-independent ways to regulate mTOR (rapamycin for one), and I think it is an oversimplification to consider mTOR as part of the "insulin/IGF-1 pathway". I would

suggest changing the title/abstract to reflect the importance of mTOR specifically and revising the text in the relevant places.

We appreciate the helpful advice and the overall positive evaluation of our manuscript. We have carefully revised the text of the manuscript as suggested. It is important to note that the genetic experiments provided in this paper clearly show a surprising interaction of insulin/IGF1 receptor signaling and the PHB2-mediated mitochondrial dysfunction. To our knowledge, this is the first paper that shows that mitochondrial dysfunction can be counteracted by gene deletion of the insulin and IGF-1 receptor in mammals. Although the reviewer very correctly states that mTOR may be regulated through various pathways our data clearly indicate that the insulin/IGF-1 receptor clearly play a role. Our data suggest that the observed effect on mTOR signaling is mediated via the insulin/IGF-1 receptor pathway which ultimately results in a survival benefit of $Phb2^{pko}$/$Insr^{pko}$/$Igf1r^{pko}$ mice. Since we also thought that the reviewer's concern is very important we added further experimental evidence and treated *Phb2*-deficient podocytes with BMS 536924 (from Tocris), an inhibitor specific for the insulin and IGF-1 receptors. This compound has been widely used to study insulin/IGF signaling in mammalian tissues and the concentrations of BMS that we chose was comparable to the published data (Wahner, Hendrickson et al, 2009; Wittman et al, 2005). Very similarly to the *in vivo* data, treatment of *Phb2*-deficient podocytes with this inhibitor prevented hyperphosphorylation of S6 ribosomal protein induced by PHB2 deficiency supporting a direct link between mitochondrial dysfunction, hyperactive IR/IGF1R signaling and downstream mTOR hyperactivity. This data is now included in new Fig. E6B. We have carefully rephrased our manuscript but decided to keep the title based on these observations.

2. I found the data in Fig E5 on DAF-16 relocalization to be somewhat distracting and not particularly relevant. It's not clear what this is actually reflecting or that the mechanisms are at all related to mTOR hyperactivation in the phb2 deficient mice. The worm data is not needed to further support the mouse data, as the P-S6 is clear indication for mTOR hyperactivation.

We appreciate the reviewer's concern and have carefully rephrased our manuscript to better explain this data point. As suggested by the reviewer, we have deleted this figure from the main body of the manuscript and present the data in supplemental Figure E7.

The relevant paragraph in the paper now reads: "These data raise the possibility that mitochondrial dysfunction resulting from *Phb2* deficiency causes hyperactive insulin/mTOR signaling in podocytes. This hypothesis was further supported by experiments in *C. elegans.* Heat shock treatment of worms results in the nuclear translocation of DAF-16 independent of insulin signaling (Figure E7A). Cytoplasmic redistribution, a process known to be controlled by insulin receptor (DAF-2) signaling, was used as an indicator of insulin receptor (DAF-2) signaling in PHB-2 proficient and deficient worms (Figure E7B-D). Consistent with hyperactive insulin signaling in PHB-2 deficient animals, loss of *phb-2* accelerated recovery of the DAF-2-dependent cytoplasmic localization of DAF-16 after heat-shock (Figure E7A-D) further supporting a role for insulin receptor mediated mTOR hyperactivity resulting from the mitochondrial defects. Taken together, these data suggested the intriguing possibility that the severe kidney disorder in $Phb2^{pko}$ mice may be amenable to a therapeutic intervention with mTOR inhibitors."

3. Similar hyperactivation of mTOR in mitochondrial dysfunction mouse and zebrafish models has been previously reported by Johnson et al. [1] and Kim et al. [2]. This should be mentioned somewhere in the text.

We thank Referee #3 for bringing the important publication by Kim et al. to our attention. Both citations are now incorporated into the text of the manuscript.

4. Some of the survival curves in Fig 4 are hard to see due to the colors used. The mortality kinetics for the phb2 insr igf1r animals are interesting, as it appears that about half of the animals obtained little benefit and half a very large benefit. Any idea what this means?

According to the reviewer's suggestion we changed the colors in the survival curves in Fig. 4. It remains unclear why some animals obtained just a little and others a very large survival benefit.

To rule out the possibility of an inefficient Cre activity when deleting three genes *in vivo* we added additional experiments. We purified genomic DNA from isolated glomeruli from 2 weeks old *Phb2*^{pko} and *Phb2*^{pko}/*Insr*^{pko}/*Igf1r*^{pko} mice and performed quantitative PCR specifically detecting the deleted *Phb2* allele. Quantitative assessment of *Cre* recombinase efficiency in *Phb2*^{pko} as compared to *Phb2*^{pko}/*Insr*^{pko}/*Igf1r*^{pko} mice revealed comparable gene deletion (new Fig. 5B) clearly indicating that differences in gene deletion efficiency cannot account for the altered mTOR activity or milder phenotype in triple knock-out animals.

5. Johnson et al. observed much greater effects on survival in the Ndufs4 KO mice at 8 mg/kg/day rapamycin compared to half that dose [1]. I wonder if much larger effects would also be seen in this model at a similar dose of rapamycin. It's not necessary to do this experiment, as the effect is clear even at the lower dose used here, but perhaps should be noted in the discussion.

In our study, we injected rapamycin at a final dosage of 3 mg/kg/day as published by Zeng et al. (Zeng *et al*, 2008), who also started the drug treatment 14 days after birth. In contrast to this approach, Johnson et al. started their injections one week later administrating much higher doses (8 mg/kg/day). We cannot exclude an even stronger effect on survival when treating the *Phb2*^{pko} mice with higher doses of rapamycin. However, as seen in Figure E8 the rapamycin dose used in our study proved to be effective in blocking the phosphorylation of S6 ribosomal protein within the entire kidney.

References for Referee #3:

Wahner Hendrickson AE, Haluska P, Schneider PA, Loegering DA, Peterson KL, Attar R, Smith BD, Erlichman C, Gottardis M, Karp JE, Carboni JM & Kaufmann SH (2009) Expression of insulin receptor isoform A and insulin-like growth factor-1 receptor in human acute myelogenous leukemia: effect of the dual-receptor inhibitor BMS-536924 in vitro. *Cancer Res.* **69:** 7635–7643

Wittman M, Carboni J, Attar R, Balasubramanian B, Balimane P, Brassil P, Beaulieu F, Chang C, Clarke W, Dell J, Eummer J, Frennesson D, Gottardis M, Greer A, Hansel S, Hurlburt W, Jacobson B, Krishnananthan S, Lee FY, Li A, et al (2005) Discovery of a (1H-benzoimidazol-2-yl)-1H-pyridin-2-one (BMS-536924) inhibitor of insulin-like growth factor I receptor kinase with in vivo antitumor activity. *J. Med. Chem.* **48:** 5639–5643

Zeng L-H, Xu L, Gutmann DH & Wong M (2008) Rapamycin prevents epilepsy in a mouse model of tuberous sclerosis complex. *Ann. Neurol.* **63:** 444–453

2nd Editorial Decision 03 June 2014

Thank you very much for sending your revised paper for peer-evaluation to The EMBO Journal editorial office. Your study has been re-reviewed by one of the previous referees (and I settled on the originally most balanced scientist). From the enclosed comments you will see that s/he recognizes the efforts and work that went into the revisions. Irrespective of this, the major criticism on a mostly descriptive dataset that not sufficiently addresses relevant mechanisms could not be resolved.

This is a serious concern, particularly in light of the aim and scope of The EMBO Journal, and I have thus no other choice than to decline further pursuit at our very selective title. Please understand that I certainly did not take this decision lightly.

With the translational aspects and value of your study having been recognized already in the first round, and the paper grown stronger during revisions, I took the liberty to inquire with one of my colleagues from EMBO Molecular Medicine. I am happy to convey that Celine Carret is very open

towards rapid pursuit for your paper at their title, considering also the existing referees comments for efficient proceedings.

Having read the paper herself, she remarked that it would need a recast of the text, away from the current basic/mechanistic premise as to re-focus on the clinical-translational implications the paper certainly carries. She would subsequently only engage a member of the editorial board as to ensure consistency and style, while warranting that no further experimental requests would be made.

I hope that this presents a valuable option for efficient presentation of your results and kindly ask you to transfer/in case of further questions get in touch with Celine directly.

REFEREE REPORT:

Referee #1:

Many thanks for asking me to re-review this paper entitled "Inhibition of insulin/IGF-1 receptor signaling protects from mitochondria- mediated kidney failure"
I appreciate much work has gone into this and the new manuscript is stronger than the initial submission. I am however concerned that the mechanism underlying the PHB2 depletion / insulin - IGF axis inhibition, and podocyte dysfunction, is still not clear. Much of this work is descriptive and still does not address the underlying mechanism. I appreciate the authors have performed multiple experiments analyzing mitochondrial function and show not difference between the PHB2 knockout and the PHB2 KO/ insulin IGF KO. It therefore does not, to me, reveal the mechanism underlying these effects. I appreciate that the link with this protein and the slit diaphragm requires more work but I still think could be critical for this albuminuric phenotype, especially as the new data suggests that insulin and IGF do not seem to alter the mitochondrial damage in this model.

Further comments:

1. I think figure 5 is helpful but would be improved if the normal level of Phb2 in control mice (without cre recombinase) was shown. This may give an idea of the amount of knockdown being achieved by the system.
2. Further analysis of figure 4 shows that IR het knockdown seems to give more survival benefit than the homozygous knock down when crossed with PHB2. There is an n of 4 (which may be accounted for by inadequate power) but this should be explained.

Transfer to EMBO Molecular Medicine — 02 December 2014

Thank you for transferring your manuscript to EMBO Molecular Medicine.

As discussed, we would like you to rewrite the paper to better emphasise the clinical and translational aspects. We also would like you to take the last set of comments from referee 1 into account when preparing your revision. where the information is to be found in the main text.

I look forward to reading a new revised version of your manuscript as soon as possible.

2nd Revision - authors' response — 16 December 2014

Thanks very much for the opportunity to submit our work to EMBO Molecular Medicine. We have worked on the manuscript and the figures and included all the required changes in the text.

Attached with this submission you will find the requested files and modified figures. Please let me know what you think.

I am looking forward to hear from you.

3rd Editorial Decision 18 December 2014

Thank you for the transfer and reformatting of your revised manuscript for EMBO Molecular Medicine. I have now heard from our editorial advisor, and I am pleased to inform you that we will be able to accept your manuscript pending editorial minor amendments.

Please submit your revised manuscript within two weeks. I look forward to seeing a revised form of your manuscript as soon as possible.
